# A single Markov-type kinetic model accounting for the macroscopic currents of all human voltage-gated sodium channel isoforms

**DOI:** 10.1371/journal.pcbi.1005737

**Published:** 2017-09-01

**Authors:** Pietro Balbi, Paolo Massobrio, Jeanette Hellgren Kotaleski

**Affiliations:** 1 Department of Neurorehabilitation, Scientific Institute of Pavia via Boezio IRCCS, Istituti Clinici Scientifici Maugeri SpA, Pavia, Italy; 2 Department of Informatics, Bioengineering, Robotics and System Engineering (DIBRIS), University of Genova, Genova, Italy; 3 Department of Neuroscience, Karolinska Institute, Stockholm, Sweden; 4 Department of Computational Science and Technology, School of Computer Science and Communication, KTH The Royal Institute of Technology, Stockholm, Sweden; Rush University Medical Center, UNITED STATES

## Abstract

Modelling ionic channels represents a fundamental step towards developing biologically detailed neuron models. Until recently, the voltage-gated ion channels have been mainly modelled according to the formalism introduced by the seminal works of Hodgkin and Huxley (HH). However, following the continuing achievements in the biophysical and molecular comprehension of these pore-forming transmembrane proteins, the HH formalism turned out to carry limitations and inconsistencies in reproducing the ion-channels electrophysiological behaviour. At the same time, Markov-type kinetic models have been increasingly proven to successfully replicate both the electrophysiological and biophysical features of different ion channels. However, in order to model even the finest non-conducting molecular conformational change, they are often equipped with a considerable number of states and related transitions, which make them computationally heavy and less suitable for implementation in conductance-based neurons and large networks of those. In this purely modelling study we develop a Markov-type kinetic model for all human voltage-gated sodium channels (VGSCs). The model framework is detailed, unifying (i.e., it accounts for all ion-channel isoforms) and computationally efficient (i.e. with a minimal set of states and transitions). The electrophysiological data to be modelled are gathered from previously published studies on whole-cell patch-clamp experiments in mammalian cell lines heterologously expressing the human VGSC subtypes (from Na_V_1.1 to Na_V_1.9). By adopting a minimum sequence of states, and using the same state diagram for all the distinct isoforms, the model ensures the lightest computational load when used in neuron models and neural networks of increasing complexity. The transitions between the states are described by original ordinary differential equations, which represent the rate of the state transitions as a function of voltage (i.e., membrane potential). The kinetic model, developed in the NEURON simulation environment, appears to be the simplest and most parsimonious way for a detailed phenomenological description of the human VGSCs electrophysiological behaviour.

## Author summary

A unifying novel kinetic model of human voltage-gated sodium channels is proposed, which is able to reproduce in detail the macroscopic currents of all the ion-channel isomers, from Na_V_1.1 to Na_V_1.9. Its topology consists of six states (two closed, two open, two inactivated) and twelve transitions, and it is particularly well suited to be implemented in biologically inspired multi-compartmental neural cells and neural network models. It represents the most parsimonious kinetic model able to account for the most recently described electrophysiological features, and it has been developed by taking into account the experimental data gathered by published work reporting on each different isomer heterologously expressed in mammalian cell lines. Equipped with original differential equations, the model reproduces in detail the ion-channel macroscopic electrophysiological features with the minimal computational load.

## Introduction

In computational neuroscience, modelling of ionic channel behaviour represents a fundamental step to develop biophysically detailed neuron models. As key players in the mechanisms underlying excitability, impulse conduction and signal transduction, both the voltage-gated and ligand-gated ion channels are essential components of the electrophysiological behaviour of each neuronal cell and, consequently, of the neural networks these cells make up [1–2].

Until recently the phenomenological behaviours of the voltage-gated ionic channels have been mainly modelled according to the formalism introduced by the seminal and forward looking work of Hodgkin and Huxley [3]. By exploiting their substantially fair approximation to the macroscopic currents of the voltage-gated ionic channels, the models derived by the Hodgkin-Huxley (HH) equations have been instantiated, even recently, in a multiplicity of realistic cellular and network models [4–7].

The overall simplicity and the relative light computational load of the HH formalism especially make them particularly well suited in modelling biologically detailed neural networks.

However, following the continuing achievements in the biophysical and molecular comprehension of these pore-forming transmembrane proteins, the HH formalism turned out to carry limitations and inconsistencies in reproducing in detail the ion-channels electrophysiological behaviour [8–12].

More detailed insights into the single channel kinetics provided by patch-clamp techniques [12–13] and into their molecular structure by means of x-ray crystallography [14] have greatly advanced our comprehension of ion channels to a degree difficult even to conceive when Hodgkin and Huxley developed their impressive and seminal research.

The more information about ion-channel gating has been achieved, the clearer is the need for models with explicit representation of single ion-channel states. In the HH formalism the gating parameters do not represent specific kinetic states of ion channels, and the HH model is sometimes not sufficient to capture various aspects of the channel behavior [15–16].

For the aforementioned reasons, Markov-type kinetic models have been developed to accurately represent an ionic channel as a collection of states and a set of transitions between them.

In recent years, many kinetic models have been developed, specifically focused on single isoforms of different channels or on specific details of them, which are derived from both functional and structural studies (e.g., [17–19]). The developed models, biophysically detailed and aimed at representing the molecular conformational changes of the transitions between states, usually carry a considerable number of states and related transitions. They are well suited to describe the detailed microscopic behaviour of single ion channels, but their computational load makes them much less suitable for the implementation in multi-compartmental conductance-based biologically detailed neurons and neural networks models.

As a result, although a variety of Markov-type kinetic models have been used to analyze the functional biophysical properties of single ion channels, yet very little of this information is used to develop conductance-based models of neural structures [20].

Thus, nowadays the two mutually exclusive options in modelling ionic channels rely on the HH formalism, which is global and computationally light, or the Markov-type kinetic models, which are specific, detailed and computationally heavy.

Among the ionic channels, voltage-gated sodium channels (VGSC) are probably the most studied and modelled voltage-gated ionic channels. They are directly involved in the cellular excitation and in the onset of the spike. In humans, nine subtypes or isomers (Na_V_1.1 to Na_V_1.9) of VGSC exist, each of them with peculiar kinetics and tissue distribution [1–2]. They exhibit a diversified and complex, membrane potential-dependent gating behavior [8], and even slight modifications of their gating kinetics by genetic mutations give rise to a number of severe human diseases in peripheral nerves, skeletal muscles, the heart and the central nervous system [2, 21].

This purely modelling study is aimed at developing a Markov-type kinetic model for VGSCs, which is detailed (accounting for different features of the VGSCs macroscopic current), unifying (accounting for all ion-channel isoforms) and computationally efficient (with a minimal set of states and transitions).

By exploiting experimental data gathered from previously published electrophysiological studies from different laboratories investigating single isomers of the VGSC, we derived a simplified common kinetic model for VGSCs, suitable to be adopted in biologically detailed simulation of neural structures.

The obtained results show that it is possible to develop a unifying kinetic model for VGSCs macroscopic currents by adopting a new simplified state diagram and novel equations describing the voltage dependence of the state transitions.

## Methods

### Experimental datasets

As we were mainly interested in modelling the macroscopic electrophysiologic behaviour of single isoforms of the sodium channel, we searched the literature for experimental data from single human VGSC (Na_V_1.1 to Na_V_1.9) α-subunits, heterologously expressed in mammalian cell line (usually Human Embrionic Kidney 293 cells) [22–29]. Studies on Na_V_1.8 and Na_V_1.9, whose transfection in non-nervous cell line is practically challenging, were performed in homozygous Na_V_1.8-cre mice Dorsal Root Ganglia neurons lacking endogenous Na_V_1.8 [30], and, respectively, in ND7/23 cells (a hybrid from mouse neuroblastoma and rat neurons) [31].

In some of the considered studies one or two β-subunits were also co-transfected: β1-subunit was coexpressed with Na_V_1.7 [29], and β3-subunit with Na_V_1.3 [25]; β1- and β2-subunits were both coexpressed in Na_V_1.1 [22] and in Na_V_1.2 experiments [23]. The electrophysiological experiments were conducted by means of the whole-cell patch-clamp method, usually at room temperature and our model corrected for the experimental temperature (by means of the temperature coefficient, Q_10_).

Modelled graphics of every VGSC with reference to the experimental ones are displayed in a Supporting Information file (S1 Appendix. Modelled graphics with reference to the original ones).

### Simulation protocols

**Fig 1:**
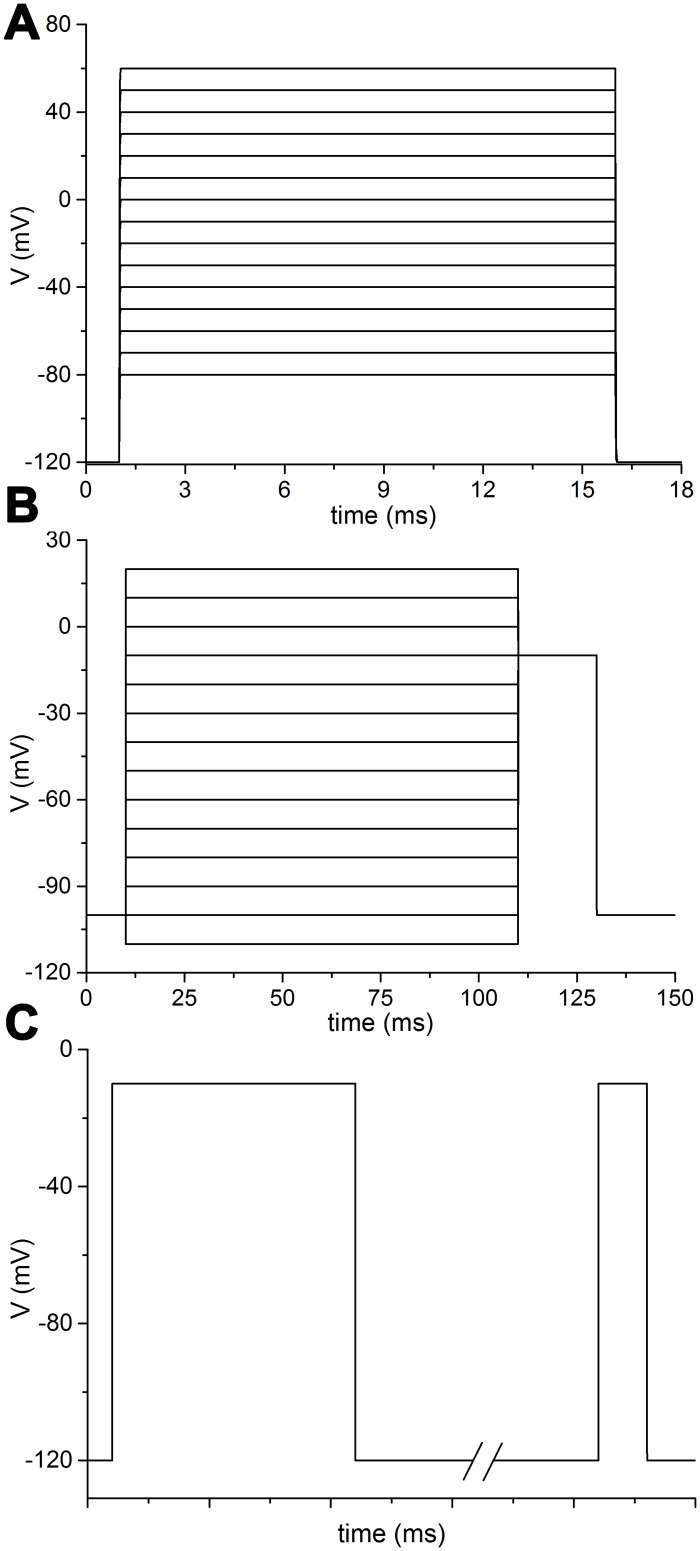
Electrophysiological protocols commonly used in the considered studies. A: Series of depolarizations generated from a holding potential, ranging from less to more depolarized values, used for recording current-voltage curves, voltage-peak relationship, conductance-voltage relationship. B: Conditioning (pre-pulse) long depolarizations at different voltages followed by a test depolarization, used for studying the voltage dependence of steady-state inactivation (availability). C: A conditioning long depolarization followed by repolarizations of increasing duration, before the test depolarization, used for studying the recovery from inactivation (repriming).

For each VGSC isomers, the following electrophysiological data were gathered and in turn reproduced by modelling:

a) Current-voltage curves, usually obtained with sequential voltage clamps in steps of 5 or 10 mV from a certain resting value (Fig 1A).
b) Current-voltage relationship, which plots the current peak values against the level of voltage clamp.
c) Normalized conductance-voltage relationship, obtained by converting the current peak values above described into the respective conductance values, according to the Eq (1),
  $$G=I/\left(V-V_{Na}\right)$$ (1)
  (where *V* is the membrane voltage, *V*_*Na*_ the sodium equilibrium potential, *V-V*_*Na*_ the driving force, *G* the conductance and *I* the peak current). The so evaluated conductance was plotted against voltage clamp values, and the conductance-voltage curves were fitted to a Boltzmann Eq (2) as below,
  G/Gmax=(1+eV−V12k)−1 (2)
  where *G*_*max*_ is the maximum conductance, *V*_*1/2*_ the half maximal voltage and *k* the slope factor.
d) Normalized current-voltage relationship during steady-state fast inactivation, also known as steady-state availability-voltage relationship (Fig 1B). The steady-state inactivation curve was fitted to a Boltzmann Eq (3) as below,
  I/Imax=A+(1−A)⋅(1+eV−V12k)−1 (3)
  where *I* is the peak current, *I*_*max*_ the maximal peak current, *A* is the fraction of non-inactivating channels, *V*_*1/2*_ the half availability voltage, and *k* the slope factor.
e) Normalized recovery from inactivation (or repriming) (Fig 1C). Recovery curves were fitted to the bi-exponential Eq (4) as below,
  $$I/I_{max}=A_{1}\left(1-e^{-\frac{t}{\tau_{1}}}\right)+A_{2}\left(1-e^{-\frac{t}{\tau_{2}}}\right)+A_{3}$$ (4)
  where *A*_*1*_ and *A*_*2*_ are the proportional coefficients, *A*_*3*_ the proportion of non-inactivating channels (if present), *t* the time, *τ*_*1*_ and *τ*_*2*_ the fast and slow recovery time constants, respectively.

When available, we also compared our simulations with the following electrophysiological data:

f) First and second time constant of fast inactivation (current decay from the transient peak of activation).
g) Time constant of deactivation, which elucidates the kinetics of open-closed states transition.
h) Onset (or development) of slow inactivation.
i) Normalized current-voltage relationship during slow inactivation.
j) Normalized recovery from slow inactivation.

### Modelling formalism

The ionic channel current is governed by Ohm's law, wherein conductance is determined by the fraction of channels in the open states, *x*_*O*_ (0 to 1):

$$I_{Na}\left(t\right)={\bar{g}}_{Na}\cdot x_{O}\left(t\right)\cdot \left(V\left(t\right)-V_{Na}\right),$$ (5)

where ${\bar{g}}_{Na}$ is the sodium maximal conductance and *V*_*Na*_ is the reversal potential of that ion. Transitions between the O (open), I (inactivated), and C (closed) states are described by conventional Markovian model equations [32] written for the fractions of channel, *x*_*O*_, *x*_*I*_, *x*_*C*_, to be in these states:

$$\frac{dx_{O}}{dt}=A_{CO}x_{C}-\left(A_{OC}+A_{OI}\right)x_{O}$$ (6)

$$\frac{dx_{I}}{dt}=A_{OI}x_{O}-A_{IC}x_{I}$$ (7)

$$x_{O}+x_{C}+x_{I}=1$$ (8)

where *A*_*XY*_ is the transition rate between the state *X* and the state *Y*. The topology of the single, general model and the transitions between its states have been searched for through progressive appoximations using heuristic optimization.

### Simulations of the experimental procedure in NEURON

All simulated experiments were performed by means of NEURON version 7.4 simulation environment [33]. The kinetic equations were written and solved directly using KINETIC methods of NMODL language of NEURON, which is a derivative of the MODL description language of the SCoP package [34].

All virtual experiments were performed on an one-compartmental cylindrical 'soma' 50 *μm* long with a diameter of 63.66 *μm*, so that the membrane area was set to 10'000 *μm*^*2*^. The membrane capacitance was set at 1 *μF/cm*^*2*^ [35]. The maximal conductance density for each VGCS isomer inserted into the soma was arbitrarily set to 0.1 *S/cm*^*2*^, and the resulting ionic current density was measured in *mA/cm*^*2*^. The capacitive currents were subtracted from the total current in all the simulations. The time for single integration step (*dt*) was set to 0.025 ms.

At every step, the rate constants of each transition were multiplied by the temperature coefficient, Q_10_, calculated as follows:

$$Q_{10}=3^{\left(\frac{T{}^{\circ}-20{}^{\circ}}{10{}^{\circ}}\right)}$$ (9)

Original NEURON source code was developed to simulate the protocols needed to yield the electrophysiological features of the channels. The simulated voltage-clamp protocols are depicted in Fig 1.

The source code along with the virtual experimental procedures is provided and available as a ModelDB [36] entry (http://modeldb.yale.edu/230137).

All simulations were performed on an iMac desktop computer running a MacOS version 10.12.5 (^™^ and © 1983–2017, Apple Inc, Cupertino, CA, USA).

### Fitting curves procedure

The developed code automatically supplied the appropriate graphics, which replicated the macroscopic currents and the electrophysiological relationships found in the experimental studies (see S1 Appendix. Modelled graphics with reference to the original ones). A both empirical and quantitative curve fitting method was then adopted to reconcile experimental and modelled data. Firstly, the curves and relationships obtained by the simulations were compared by visual inspection to the experimental ones. Then, the modelled curves were fitted for the Eqs (2) to (4), as appropriate, by using a nonlinear least-squares minimization method included in NEURON (Multiple Run Fitter subroutine), which in turn derives from the PRAXIS (principal axis) method described by Brent [37]. Finally, the parameters of the Eqs (2) to (4) of the modelled curves were compared to the experimental ones (Table 1). The agreement of the modelled data with the experimental ones was considered acceptable when the former were within two standard deviations of the latter.

**Table 1:** Comparisons between experimental and simulated electrophysiological values.

Electrophysiological parameters		Na_V_1.1	Na_V_1.2	Na_V_1.3 $	Na_V_1.4	Na_V_1.5	Na_V_1.6	Na_V_1.7 §	Na_V_1.8	Na_V_1.9 &
TC HMA of normalized conductance (mV)	Exp.	-23.6 ± 1.2	-25.3 ± 1.4	-24.1 ± 0.9	-23.7 ± 1.2	-34.5 ± 1.5	-29.2 ± 1.8	-36.15 ± 1.23	-1.11 ± 1.6	-56.9 ± 0.6
	**Model**	**-24.1**	**-25.6**	**-24.5**	**-24.0**	**-33.9**	**-29.6**	**-36.28**	**-1.3**	**-56.3**
Slope of normalized conductance		-7.4 ± 0.3	-7.5 ± 0.4	-7.8 ± 0.1	-7.7 ± 0.2	-7.2 ± 0.6	-6.0 ± 0.2	-6.66 ± 0.34	-8.77 ± 0.45	-6.7 ± 0.3
		**-7.0**	**-7.3**	**-7.8**	**-7.8**	**-7.3**	**-6.2**	**-6.46**	**-8.09**	**-6.9**
SS HMI of normalized current during fast inactivation (mV)		-64.2 ± 1.1	-67.4 ± 1.7	-71.9 ± 1	-75.9 ± 1.6	-89.1 ± 1.6	-71.6 ± 2 *	-93.60 ± 1.26	-29.9 ± 1.2	-52.1 ± 2.6
		**-63.7**	**-67.2**	**-72.1**	**-76.6**	**-89.2**	**-71.5** *	**-93.40**	**-30.3**	**-52.5**
Slope of normalized current during fast inactivation		5.8 ± 0.1	9.1 ± 0.8	7.4 ± 0.3	7.2 ± 0.4	5.5 ± 0.4	6.5 ± 0.3	4.94 ± 0.24	6.33 ± 0.30	9.2 ± 0.7
		**5.9**	**9.1**	**7.7**	**7.2**	**5.0**	**6.3**	**4.68**	**6.0**	**9.8**
First time constant of the recovery from inactivation (ms)		3.8 ± 0.5	1.4 ± 0.1	13 ± 2	2.3 ± 0.2	5.1 ± 0.9 **	12.6 ± 2	9.54 ± 2.00	3.97 ± 0.91	13.6 ± 2.2
		**3.8**	**1.5**	**13.2**	**2.3**	**5.3**	**12.4**	**9.47**	**4.06**	**13.2**
Fraction of first recovery from inactivation (%)		83 ± 2	75 ± 2	-	84 ± 4	78 **	-	-	-	52.3 ± 7.3
		**83**	**76**		**83**	**78**				**37.0**
Second time constant of the recovery from inactivation (ms)		96 ± 16	53.6 ± 6.9	-	113 ± 43	596.3 **	-	-	-	77.9 ± 11.5
		**122**	**53.6**		**116**	**602.3**				**76.6**
Fraction of second recovery from inactivation (%)		17 ± 2	24 ± 2	-	16 ± 4	22 **	-	-	-	24.4 ± 6.9
		**17**	**24**		**17**	**22**				**28.5**
Reference		[22]	[23]	[24, 25]	[26]	[27]	[28]	[29]	[30]	[31]

TC: transient current; HMA: hemi-maximal activation; SS: steady-state; HMI: hemi-maximal inactivation; Exp.: experimental

^$^ Experimental values from wild-type Nav1.3 without β3 subunit co-expression.

^§^ Experimental data from the 5N11S splice variant.

^&^ Experimental data following incubation of transfected cells at 28°C.

* Values corresponding to the conditioning pulse of 1-s duration. By adopting duration of 10 ms of the conditioning pulse, the experimental values (Burbidge et al, 2002) are -53 ± 2 mV (SS HMI) and 11.6 ± 0.6 (slope), respectively; the corresponding simulated values are -56.0 mV and 9.0.

** Values corresponding to the conditioning pulse of 1-s duration.

### Implementation samples

In order to merely test the suitability of our model to be implemented in cell models, and to compare the features of the spikes it provides to those carried out by other channel models (built according to both HH or Markov-type formalisms), we inserted the developed channel model in three previously published cell models [11, 38–39] and performed a series of voltage-clamp and current stimulation simulations.

The previously published cell models were downloaded from the ModelDB [36] repository and are accessible, respectively, with the accession numbers: 3805, 98005, 180370. For model specifications see the corresponding papers [11, 38–39].

The first implementation sample, where our model is compared to a sodium channel model developed according to the HH formalism, is reported in the Result section. The remaining two samples are provided in a Supporting Information file (S2 Appendix. Examples of model channel implementation). The second sample compares the performances of our model with a more complex Markov-type model, the third sample shows how our model behaves in a morphologically detailed neuron model.

## Results

### The electrophysiological features

Table 1 shows the values of the main electrophysiological features reproduced by the model, alongside the available corresponding experimental values for comparison, in all VGSC subtypes. The displayed values are the parameters of the fitting of experimental and simulated curves with the Eqs (4) to (6). It can be noted that the most of the modelled data are within one standard deviation of the experimental values.

**Fig 2:**
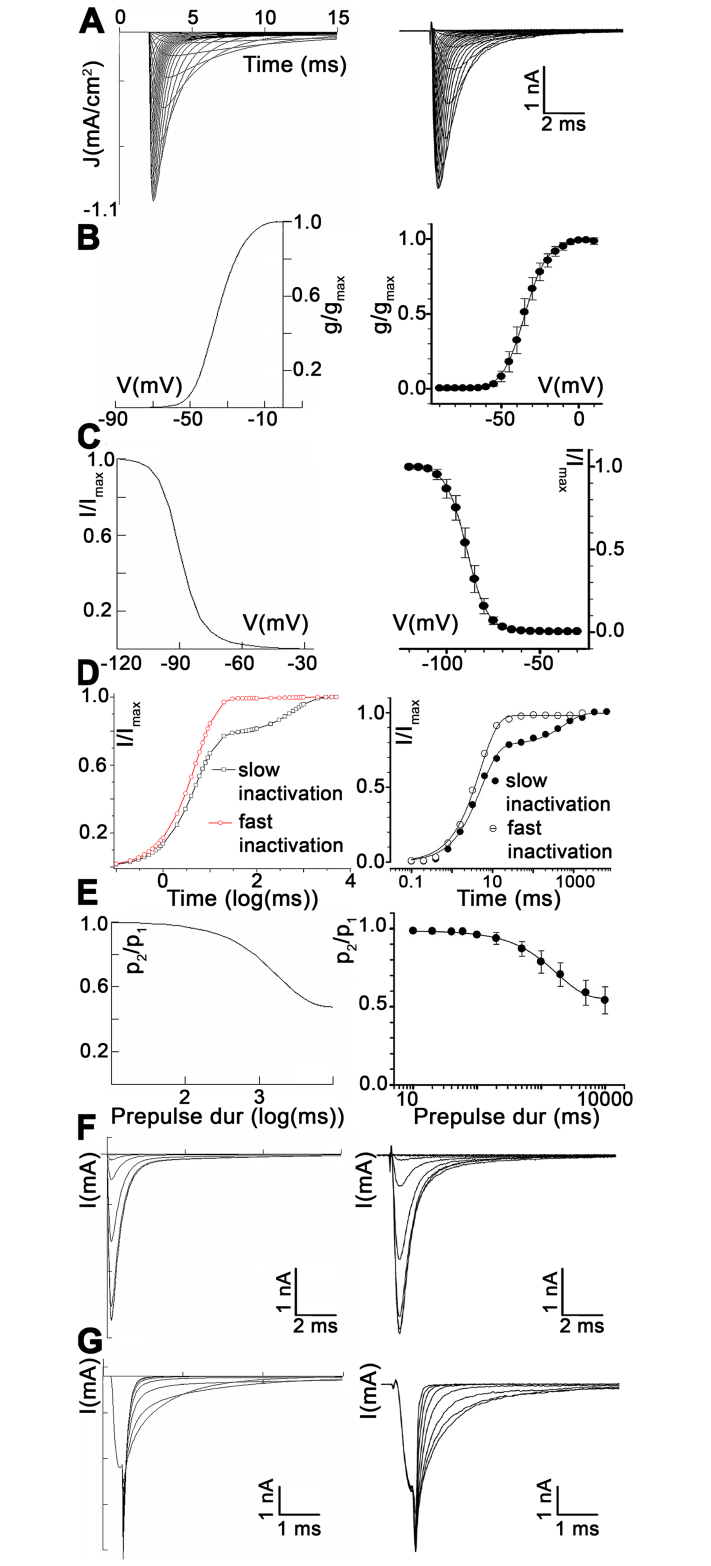
Comparison between experimental and modelled data in Na_V_1.5. A: Voltage-clamp curves from -90 mV to 60 mV in steps of 5 mV. B: Voltage dependence of the normalized conductance. C: Steady-state availability during fast inactivation. D: Recovery from fast and slow inactivation. E: Development of slow inactivation. F: Availability curves. G: Deactivation curves. Left column: modelled data. Right column: experimental data. Experimental data reproduced from [27], under the terms of the Creative Commons Attribution License.

Graphics from a single isomer, namely the Nav1.5 VGSC, comparing experimental and modelled data, are shown in Fig 2. The complete set of graphics for each VGSC isomer, showing the curves obtained during the simulations, with reference to the corresponding experimental data in previously published electrophysiological studies, is provided as a Supporting Information file (S1 Appendix. Modelled graphics with reference to the original ones).

### State diagram of the model

**Fig 3:**
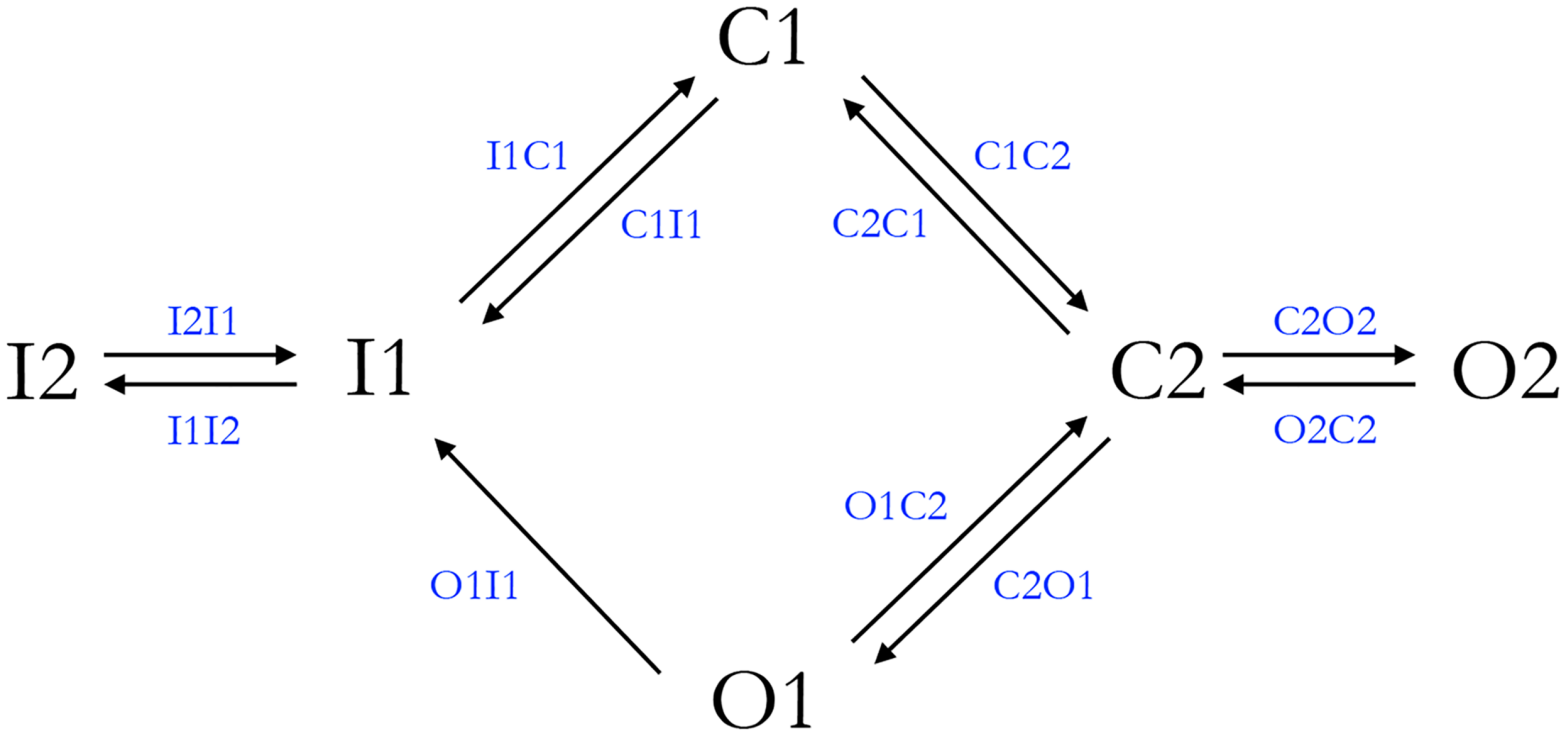
State diagram and transitions of the proposed six-state kinetic model.

The most parsimonious state diagram able to account for the phenomenological behaviour of all the VGSC isomers was found to be a six-state one (Fig 3). It is arranged in two closed, two open and two inactivated states.

The second inactivated state (I2) was considered as a deeper inactivated state than I1, only connected to I1. This topology was found to be the simplest to consistently account for both the slow and fast inactivations, as well as for the two time constants of the recovery from inactivation.

The second open state (O2), only linked to C2, was added to reproduce in detail a second slower constant of decay from activation, usually detectable on the current-voltage curves. An alternative solution, which considered O2 as only linked to O1 (by analogy with I1 and I2), was discharged because it provided not realistic tail currents of deactivation.

Two closed states (C1 and C2) were found to be sufficient to faithfully reproduce the activation kinetics and the tail currents after a brisk repolarization.

All transitions between two consecutive states were considered reversible (with one exception, see below), and the paired forward and backward transitions were computed by equations carrying numerical values (coefficients) of the same order of magnitude. The only exception was the O1 to I1 transition, where the backward transition (I1 to O1) was described by an infinitesimal value, so that the O1 to I1 transition could be considered irreversible.

The dynamics of the different states of the channels are described by the following set of coupled ordinary differential equations:

$$\frac{dC1}{dt}=I1C1*I1+C2C1*C2-\left(C1C2+C1I1\right)*C1$$ (10)

$$\frac{dC2}{dt}=C1C2*C1+O1C2*O1+O2C2*O2-\left(C2C1+C2O1+C2O2\right)*C2$$ (11)

$$\frac{dO1}{dt}=C2O1*C2+I1O1*I1-\left(O1C2+O1I1\right)*O1$$ (12)

$$\frac{dO2}{dt}=C2O2*C2-O2C2*O2$$ (13)

$$\frac{dI1}{dt}=I2I1*I2+C1I1*C1+O1I1*O1-\left(I1C1+I1I2+I1O1\right)*I1$$ (14)

$$\frac{dI2}{dt}=I1I2*I1-I2I1*I2$$ (15)

Moreover, the states obey the law of mass conservation:

O1+O2+I1+I2+C1+C2=1 (16)

### The transitions between states

**Fig 4:**
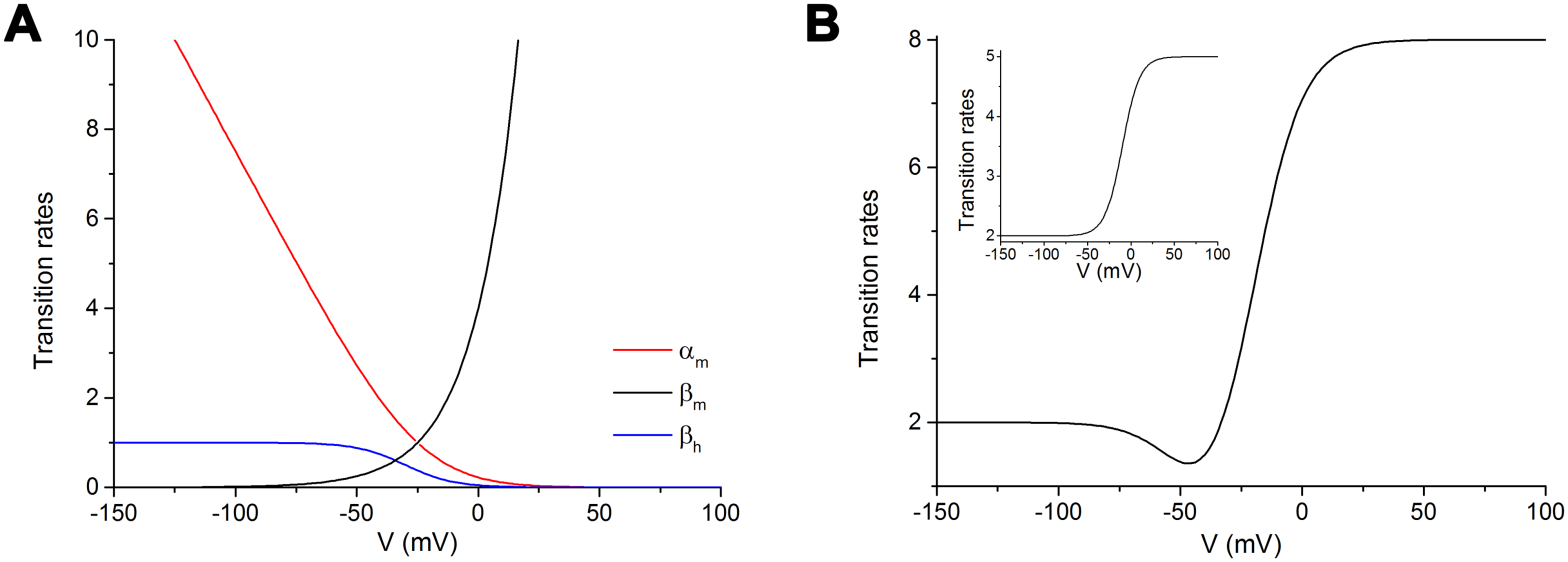
Different formal mathematical descriptions of transition rates. A: Curves drawn from the original equations by Hodgkin and Huxley [3] for the sodium channel rate constants *α*_*m*_ (red line), *β*_*m*_ (similar also to *α*_*h*_) (black line), and *β*_*h*_ (blue line). B: Modified sigmoid curve adopted in the present study (see text for details). Inset: Sigmoid curve with minimum and maximum asymptotes.

Since the studies by Hodgkin and Huxley [3], the voltage dependence of the rate transitions has been mathematically modelled (Fig 4A) as an exponential equation (black line), or as a sigmoid (blue line), or as a combined linear and exponential equation (red line).

In other cases [32, 40] a sigmoid curve with minimum and maximum asymptote has been adopted (Fig 4B inset), described by equations as below,

$$A_{\omega }=\tau_{min}^{\omega }+\tau_{max}^{\omega }\cdot {\left[1+exp\left(\frac{V-V_{1/2}^{\omega }}{k^{\omega }}\right)\right]}^{-1}$$ (17)

where *ω* is the transition between two states, $\tau_{min}^{\omega }$ and $\tau_{\text{max}}^{\omega }$ are the two asymptotes, $V_{1/2}^{\omega }$ the hemiactivation voltage, and *k*^*ω*^ the slope which describes the voltage sensitivity of the transition rate.

By progressive optimizations, we found the most suitable general equation to be adopted in all the transitions was a sigmoid one. For most of the transitions, the minimum asymptote was conveniently set to zero, while in a few cases, notably for the O1 to I1 transition, it needed a non-zero value. Furthermore, to accommodate in detail the time course of the current-voltage curves, it was found appropriate to slightly modify the sigmoid, adding a bending at the beginning of the rising slope of the curve (Fig 4B).

This way, the modified sigmoid could be mathematically described as the combination of two sigmoids with opposite slope: the first one placed towards the hyperpolarized side and carrying a positive slope factor, the second one toward the depolarized side and carrying a negative slope factor.

As a result, the general equation adopted to describe this double sigmoid was set as follows:

$$A_{\omega }=B_{hyp}^{\omega }\cdot {\left[1+e^{\left(\frac{V-V_{hyp}^{\omega }}{k_{hyp}^{\omega }}\right)}\right]}^{-1}+B_{dep}^{\omega }\cdot {\left[1+e^{\left(\frac{V-V_{dep}^{\omega }}{k_{dep}^{\omega }}\right)}\right]}^{-1}$$ (18)

where $B_{hyp}^{\omega }$, $V_{hyp}^{\omega }$ and $k_{hyp}^{\omega }$ are the magnitude, the hemiactivation and the slope factor, respectively, of the voltage dependence of the transition rate *ω* in the hyperpolarized region, and $B_{dep}^{\omega }$, $V_{dep}^{\omega }$ and $k_{dep}^{\omega }$ the corresponding values in the depolarized region.

With this formalism, the slope factor (*k*) in the hyperpolarized region assumes a positive value and a negative value in the depolarized one. In addition, when the transition rate is better described by a simple sigmoid, which happens in most of the transitions, one of the two terms of the general equation can be conveniently dropped.

The complete set of parameters values for the simulation of all the sodium channel isomers is provided in Table 2.

**Table 2:** Parameters of the rate equations for each VGSC isomer.

Na_V_1.1							Na_V_1.2							Na_V_1.3						
Transition	B(hyp)	v(hyp)	k(hyp)	B(dep)	v(dep)	k(dep)	Transition	B(hyp)	v(hyp)	k(hyp)	B(dep)	v(dep)	k(dep)	Transition	B(hyp)	v(hyp)	k(hyp)	B(dep)	v(dep)	k(dep)
C1C2				18	-7	-10	C1C2				16	-5	-10	C1C2				8	-7	-9
C2C1	3	-37	10	18	-7	-10	C2C1	3	-35	10	16	-5	-10	C2C1	2	-37	9	8	-7	-9
C2O1				18	-7	-10	C2O1				16	-10	-10	C2O1				8	-17	-9
O1C2	3	-37	10	18	-7	-10	O1C2	3	-40	10	16	-10	-10	O1C2	2	-47	9	8	-17	-9
C2O2				0.08	-10	-15	C2O2				0.13	-20	-15	C2O2				0.13	-15	-5
O2C2	2	-50	7	0.2	-20	-10	O2C2	2	-60	6	0.7	-10	-15	O2C2	1	-40	3	0.2	-20	-3
O1I1	8	-37	13	17	-7	-15	O1I1	3	-41	12	16	-11	-12	O1I1	2	-52	13	8	-22	-13
I1O1	0.00001	-37	10				I1O1	0.00001	-42	10				I1O1	0.00001	-52	10			
I1C1	0.21	-61	7				I1C1	0.55	-65	7				I1C1	0.062	-70	10			
C1I1				0.3	-61	-5.5	C1I1				0.55	-65	-11	C1I1				0.09	-68	-8
I1I2				0.0015	-90	-5	I1I2				0.0022	-90	-5	I1I2				0.0001	-90	-5
I2I1	0.0075	-90	15				I2I1	0.017	-90	15				I2I1	0.001	-90	15			
Na_V_1.4							Na_V_1.5							Na_V_1.6						
Transition	B(hyp)	v(hyp)	k(hyp)	B(dep)	v(dep)	k(dep)	Transition	B(hyp)	v(hyp)	k(hyp)	B(dep)	v(dep)	k(dep)	Transition	B(hyp)	v(hyp)	k(hyp)	B(dep)	v(dep)	k(dep)
C1C2				16	-3	-9	C1C2				10	-13	-10	C1C2				14	-8	-10
C2C1	3	-33	9	16	-3	-9	C2C1	1	-43	8	10	-13	-10	C2C1	2	-38	9	14	-8	10
C2O1				16	-8	-9	C2O1				10	-23	-10	C2O1				14	-18	-10
O1C2	1	-38	9	16	-8	-9	O1C2	1	-53	8	10	-23	-10	O1C2	4	-48	9	14	-18	-10
C2O2				0.03	-20	-8	C2O2				0.05	-10	-10	C2O2				0.0001	-10	-8
O2C2	3	-50	8	0.1	-20	-8	O2C2	2	-50	10	0.08	-20	-10	O2C2	0.0001	-55	10	0.0001	-20	-5
O1I1	0	-10	10	16	-10	-10	O1I1	7	-44	13	10	-19	-13	O1I1	6	-40	13	10	15	-18
I1O1	0.00001	-10	10				I1O1	0.00001	-20	10				I1O1	0.00001	-40	10			
I1C1	0.35	-70	10				I1C1	0.19	-110	7				I1C1	0.1	-86	9			
C1I1				0.8	-70	-7	C1I1				0.016	-92	-6	C1I1				0.08	-55	-12
I1I2				0.0015	-70	-12	I1I2				0.00022	-50	-5	I1I2				0.00022	-50	-5
I2I1	0.007	-70	12				I2I1	0.0018	-90	30				I2I1	0.0018	-90	30			
Na_V_1.7							Na_V_1.8							Na_V_1.9						
Transition	B(hyp)	v(hyp)	k(hyp)	B(dep)	v(dep)	k(dep)	Transition	B(hyp)	v(hyp)	k(hyp)	B(dep)	v(dep)	k(dep)	Transition	B(hyp)	v(hyp)	k(hyp)	B(dep)	v(dep)	k(dep)
C1C2				16	-18	-9	C1C2				5	17	-8	C1C2				0.8	-21	-9
C2C1	6	-48	9	16	-18	-9	C2C1	1	-23	8	5	17	-8	C2C1	0.05	-56	10	0.8	-21	-9
C2O1				16	-23	-9	C2O1				5	13	-8	C2O1				0.8	-61	-9
O1C2	2	-53	9	16	-23	-9	O1C2	1	-27	8	5	13	-8	O1C2	0.5	-96	10	0.8	-61	-9
C2O2				0.01	-35	-5	C2O2				0.02	15	-8	C2O2				0.0001	-5	-8
O2C2	3	-75	5	0.01	-35	-5	O2C2	0.8	-60	5	0.002	10	-6	O2C2	0.0001	-65	7	0.0001	-15	-12
O1I1	4	-52	12	8	-27	-12	O1I1	0.8	-21	10	1	-1	-7	O1I1	0.04	-59	8	0.8	1	-10
I1O1	0.00001	-52	10				I1O1	0.00001	-21	10				I1O1	0.00001	-60	8			
I1C1	0.085	-110	5				I1C1	0.28	-61	9.5				I1C1	0.06	-59	8			
C1I1				0.025	-55	-20	C1I1				0.02	-10	-20	C1I1				0.04	-59	-8
I1I2				0.00001	-80	-20	I1I2				0.001	-50	-3	I1I2				0.0016	-60	-20
I2I1	0.00001	-80	20				I2I1	0.0003	-50	5				I2I1	0.0115	-100	8			

See Eq (18) for parameters definitions.

### The activation sequence

As a general rule, according to previously proposed Markov-type channel models [e.g., 27, 41], the backward and forward transitions between two consecutive states are described by equations with opposite slopes. This arrangement is usually adopted to account for the wide differences in state occupancy of the channel at different voltage values.

**Fig 5:**
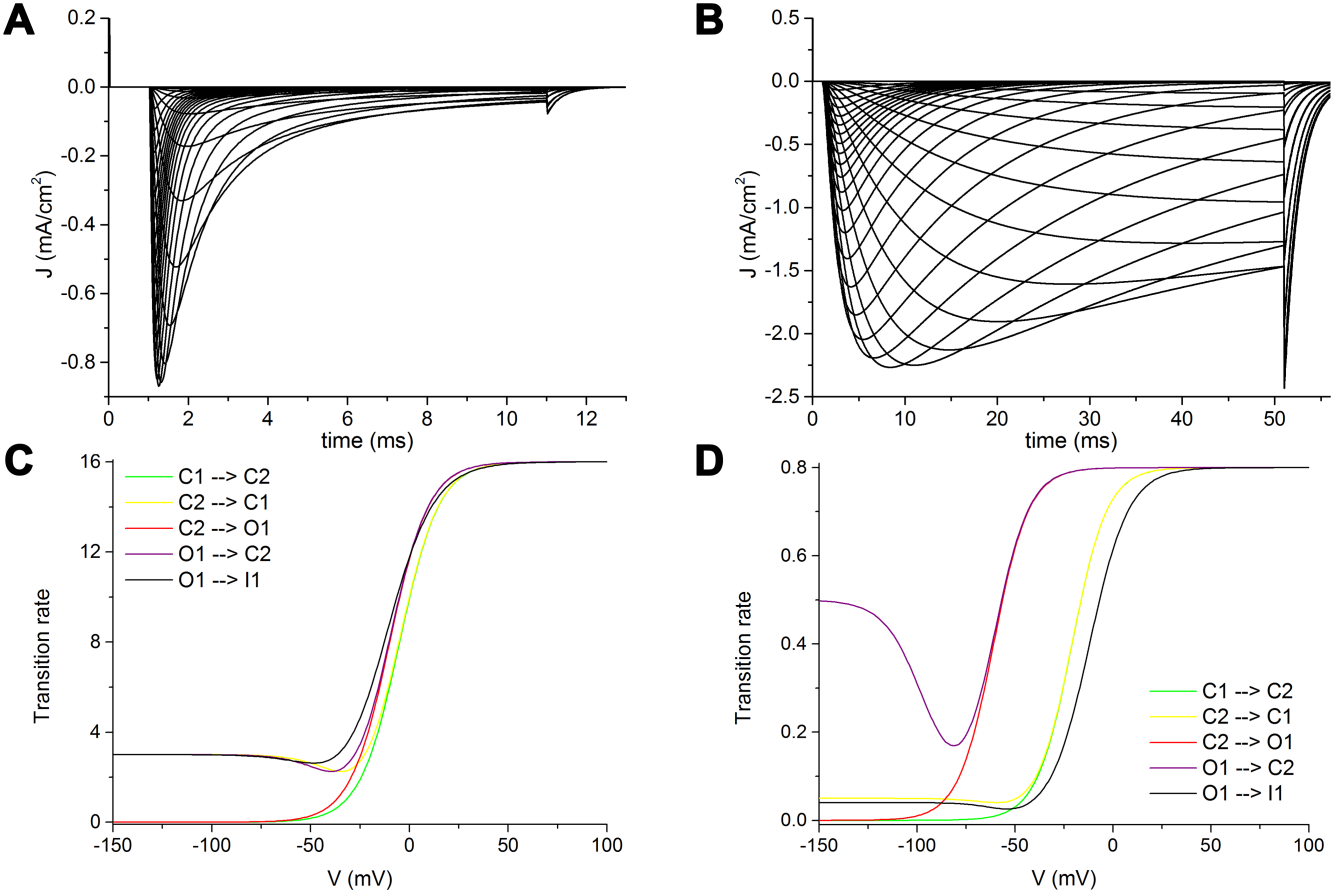
Features of activation sequence in Na_V_1.2 and Na_V_1.9. Modelled Na_V_1.2 (A) and Na_V_1.9 (B) voltage-clamp curves from -80 mV to 70 mV in 5 mV increments, from a -120 mV holding potential. Na_V_1.2 (C) and Na_V_1.9 (D) transition rates dependence from voltage of the activation sequence (comprehensive of fast inactivation). Green: C1 to C2; yellow: C2 to C1; red: C2 to O1; purple: O1 to C2; black: O1 to I1.

Yet, in modelling the voltage-intensity curves and relations, we obtained more realistic results by adopting for the activation sequence (C1 to C2 to O1 and reverse) equations with identical slopes of the main sigmoid. Moreover, in paired forward and backward transitions an identical hemivoltage point was found to consistently fit the experimental data, as well as a shift of the transitions between C1 and C2 towards more depolarized values than the transitions between C2 and O1. As an example, Fig 5 shows the voltage-intensity curves of Na_V_1.2 (Fig 5A) and Na_V_1.9 (Fig 5B). This is an instance of the most divergent electrophysiological behaviour in two channel isomers, yet the model is able to reproduce in detail the real data in both the cases. The transition rates dependences from voltage in C1 to C2 transition (green), C2 to C1 (yellow), C2 to O1 (red), O1 to C2 (purple), and O1 to I1 (black) are depicted in Fig 5C and 5D. The forward and backward transitions between two consecutive states have identical, not opposite, slope values and identical hemiactivation points. The transitions between C1 and C2 are shifted to more depolarized values compared to the transition between C2 and O1.

In addition, the backward transitions (C2 to C1, and O1 to C2) are described by a double sigmoid, whereas the corresponding forward transitions by simple sigmoids. Here the double sigmoid of the backward transitions accounts for the short time constants of deactivation recorded as tail currents (right end of the curves in Fig 5A and 5B). The higher values, indeed, of the backward transition rates at more hyperpolarized voltages drive the channel into more closed states with short latency during brisk repolarizations.

### Recovery from fast inactivation

**Fig 6:**
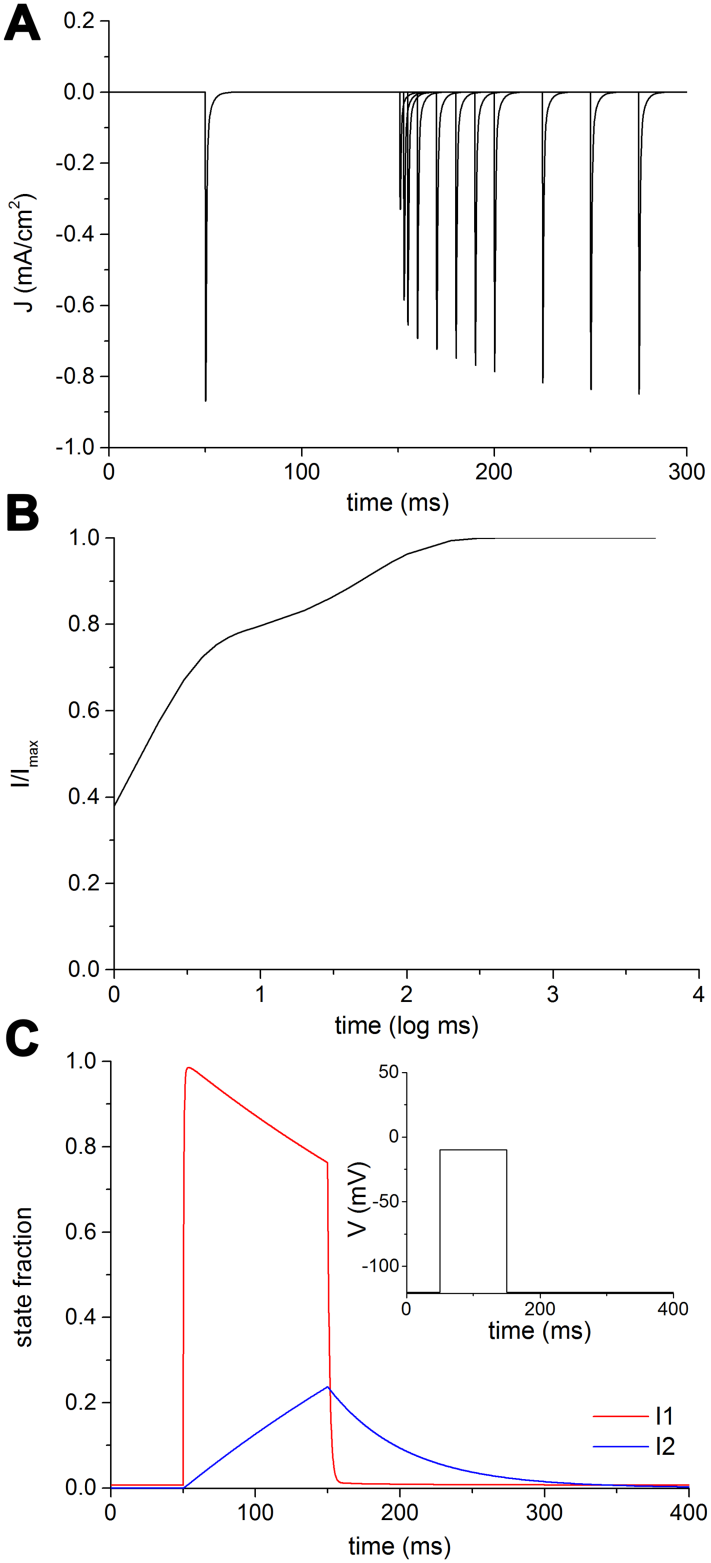
Recovery from inactivation in Na_V_1.2. A: Superimposed simulated current traces recorded with increasing duration of repolarization interval. B: Time dependent fractional recovery curve from fast inactivation. C: Fraction of channels in the inactivated states I1 (red) or I2 (blue) as brought about by the repriming protocol depicted in the inset.

Fig 6 shows the plot of the simulated results obtained during recovery from inactivation (repriming) in Na_V_1.2.

A relatively long depolarizing conditioning pulse sets all the channels up into inactivated states (Fig 1C). A following repolarizing step, variable in duration, enables the transition from inactivated to closed states (recovery from inactivation) in a proportion of channels, which increases with the duration of the repolarizing step. The subsequent depolarizing test pulse probes the proportion of the channels having recovered from the inactivation. The progressive recovery with increasing duration of the repolarization is shown in Fig 6A. In the graphic of Fig 6B, the relative amplitude of the transient current following the test pulse is drawn against the logarithm of the duration of the repolarizing step. The fast and slow time constants of recovery depend on the interplay between the two inactivated states I1 and I2 during the first depolarizing step and the following repolarizing phase. As can be seen in Fig 6C, where the fractions of I1 (red line) and I2 (blue line) states are plotted against time, a step depolarization (inset) suddenly drives almost all channels into I1 state. As the depolarization lasts (100 ms in this simulation) an increasing fraction of channels slowly moves to the I2 state. When repolarizing, the exit from the inactivated states follows a fast (I1, red) and a slow (I2, blue) course, which together account for the two time constants of recovery. By fine-tuning the parameters of I1 to I2 transition, and those of I2 to I1 transition, the relative fractions of inactivated states and the slow time constant of recovery, respectively, can be adjusted to consistently reproduce the experimental data.

### Other electrophysiological features

#### Decay from the peak of activation

**Fig 7:**
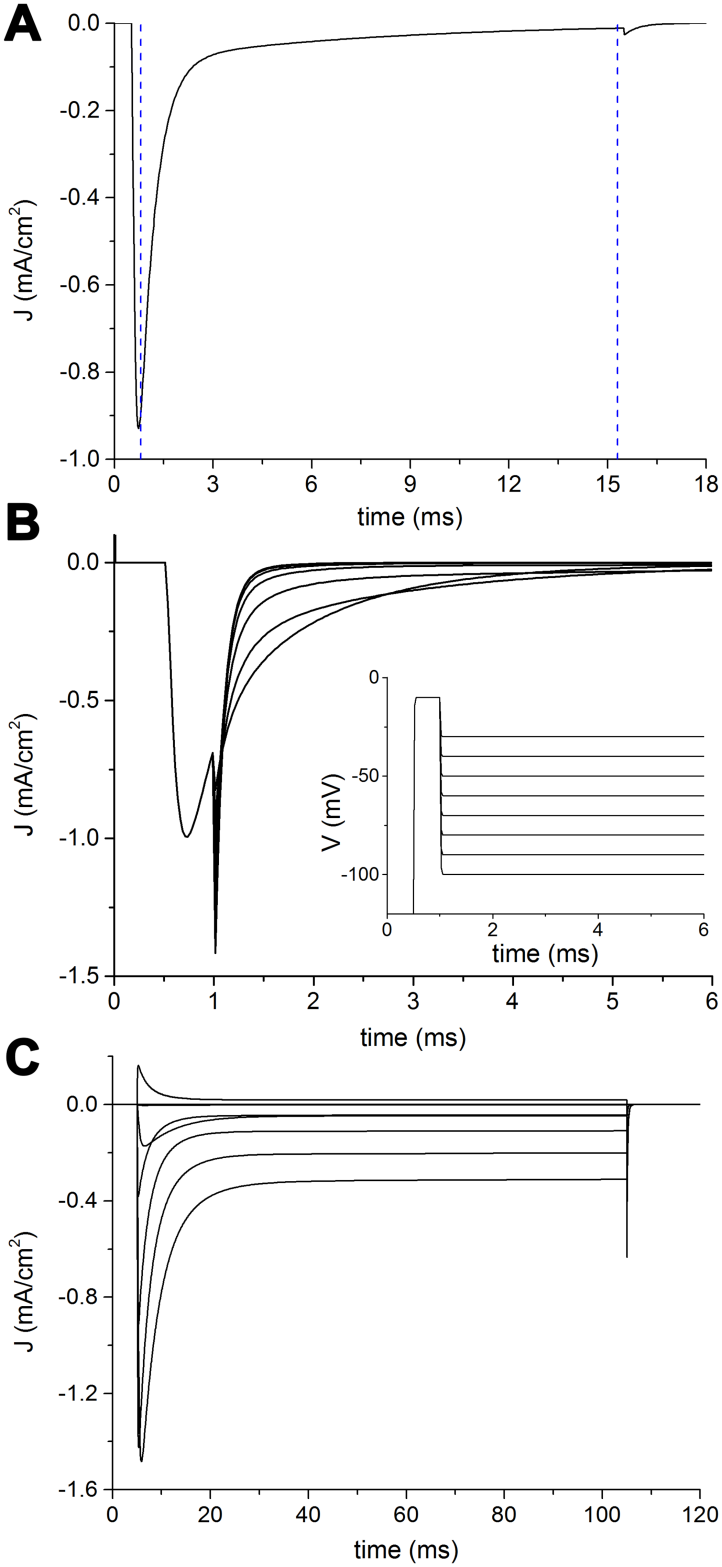
Other electrophysiological features. A: Decay from peak of activation during a voltage-clamp of -10 mV in Nav 1.1 modelled channel. The curve between the two vertical blue lines has been fitted to a double exponential (see text for details). B: Simulated deactivation curves in Na_V_1.5 following the protocol depicted in the inset. C: Simulated persistent current in Na_V_1.6.

In some of the experimental studies here considered, the decay phase from the transient peak of activation (Fig 7A) has been fitted with a double exponential function [22–23, 25]:

$$I/I_{max}=A_{1}\cdot e^{-\frac{t}{\tau_{1}}}+A_{2}\cdot e^{-\frac{t}{\tau_{2}}}+A_{3}$$ (19)

where *A*_*n*_ and *τ*_*n*_ refer to fractional amplitude and time constant, respectively. In the remaining experimental studies a single exponential function [26–31] has been used:

$$I/I_{max}=A_{1}\left(1-e^{-\frac{t}{\tau_{1}}}\right)+A_{2}$$ (20)

Table 3 shows the comparisons between the experimental data and those simulated by the proposed model. For the modelled decay curves of the isomers Na_V_1.4, Na_V_1.5 and Na_V_1.6, a better fitting was provided by the double exponential Eq (19). Thus, for these three isomers the values of the two constants and the fractional amplitude of the slow time constant obtained by the simulations are showed in Table 3.

**Table 3:** Time constants of decay from the peak of activation (recorded at a voltage clamp corresponding to the maximal peak of activation).

VGSC		*τ*_*fast*_ (ms)	*τ*_*slow*_ (ms)	fractional amplitude of *τ*_*slow*_ (%)	Reference
Na_V_1.1 *	Real	∼ 0.5	∼ 5	∼ 6	[22] Fig 2b and 2c
	**Model**	**0.52**	**5.39**	**7.3**	
Na_V_1.2		0.47 ± 0.04	3.3 ± 0.3	7 ± 1	[23] Table 2
		**0.60**	**3.4**	**9**	
Na_V_1.3		0.70 ± 0.02	4.49± 0.43	16.08 ± 1.51	[25] Table 1
		**1.02**	**5.17**	**21.41**	
Na_V_1.4 *		∼ 0.7	-	-	[26] Fig 4E
		**0.56**	**11.16**	**3.25**	
Na_V_1.5 *		∼ 0.6	-	-	[27] Fig 1F
		**0.85**	**15.95**	**4.74**	
Na_V_1.6 *		∼ 1	-	-	[28] Fig 3C
		**1.2**	**-**	**-**	
Na_V_1.7 *		∼ 0.6	-	-	[29] Fig 3A
		**0.9**	**-**	**-**	
Na_V_1.8 *		∼ 5	-	-	[30] Fig 2F
		**3.94**	**417**	**8**	
Na_V_1.9 *		∼ 70	-	-	[31] Fig 2D
		**59.95**	**-**	**-**	

* Experimental values indirectly deduced by Figures on the original papers.

In each isomer, the decay phase obviously changes according to the voltage clamp, and a relationship can be drawn between the changing voltage clamp and the time constants of decay. The curves depicting this relationship for each isomer can be found in S1 Appendix, along with the reference to the corresponding experimental curve, when available.

In the model the fast time constant of decay is mainly determined by the magnitude of the C2 to O1 and O1 to I1 transitions, the slow time constant and its fractional amplitude by the parameters of the transitions between C2 and O2.

#### Deactivation curves

Repolarizing test pulses at variable potentials following a short (usually 0.5 ms long) depolarizing pulse (Fig 7B inset) evoke the so-called tail currents (Fig 7B), useful to study the kinetics of deactivation, which are brought about by the direct and inverse transitions from open to closed states. Among the considered experimental studies, analyses of the deactivation curves are only available for Na_V_1.5 [27] and Na_V_1.7 [29] isomers.

The experimental data on time constants of deactivation compared to the modelled ones can be found in S1 Appendix.

In the proposed model, a graded regulation of deactivation kinetics is mainly achieved by tuning the transition parameters from O1 to C2.

#### Slow inactivation

Few experimental studies among the considered ones, namely those dealing with the Na_V_1.1 [22], Na_V_1.4 [26] and Na_V_1.5 [27] isomers, investigated more in detail the kinetics of slow inactivation. To this aim, the adopted electrophysiological protocols carried a longer conditioning stimulus, able to move the channels into slow inactivation states, followed by a short repolarization to allow the recovery of fast inactivation, before the test stimulus. In Na_V_1.1 and Na_V_1.4, by using extremely long conditioning pulse (30 and 10 seconds, respectively), a third ultra-slow component of inactivation emerges, with several seconds long time constant (Table 4). This ultra-slow inactivation can be accounted for by the proposed model only by adding a seventh inactivated state, deeper than I2 and only linked to I2. The comparison between experimental and simulated values of the slow and ultra-slow kinetics is shown in Table 4. At variance with the Na_V_1.1 and Na_V_1.4 experimental protocols, the Na_V_1.5 study adopts a 1000 ms long conditioning pulse. In this case, the proposed six-state model is able to account for its shorter time constants of repriming, as shown in Table 4.

**Table 4:** Experimental and simulated electrophysiological values of slow inactivation.

Electrophysiological parameters		Na_V_1.1	Na_V_1.4	Na_V_1.5
Onset				
τ_1_ (s)	Real	0.863 ± 0.113	2.2 ± 0.38	1.79 ± 0.11
	**Model**	**0.493**	**1.7**	**1.72**
Fractional amplitude of τ_1_ (%)		55 ± 5	21 ± 3	not available
		**64**	**26**	**43**
τ_2_ (s)		7.585 ± 2.352	38.84 ± 10.48	not available
		**10.678**	**27.9**	**27.89**
Fractional amplitude of τ_2_ (%)		29 ± 5	20 ± 2	not available
		**11**	**12**	**36**
Residual current		16 ± 2	59 ± 3	not available
		**25**	**62**	**21**
Voltage dependence				
V_1/2_ (mV)		-72.5 ± 1.8	-62.4 ± 2.8	not available
		**-59.7**	**-40.7**	
k		6.4 ± 0.3	11.5	not available
		**7.5**	**13.2**	
Residual current (%)		12 ± 2	69	not available
		**30**	**61**	
Recovery				
τ_1_ (ms)		228 ± 26	115 ± 8	5.2
		**122.5**	**114.9**	**5.3**
Fractional amplitude of τ_1_ (%)		67 ± 2	73 ± 2	78
		**67.8**	**74**	**80**
τ_2_ (ms)		2634 ± 246	1151 ± 144	596.3
		**2714**	**1251**	**610.4**
Fractional amplitude of τ_2_ (%)		33 ± 2	27 ± 2	22
		**32.2**	**26**	**20**
References		[22]	[26]	[27]

k = slope factor; V_1/2_ voltage of half-maximal (in-)activation; τ = time constant.

#### Persistent current

Persistent currents can be recorded in voltage-intensity curves for Na_V_1.8 and Na_V_1.9 isomers (Fig 5B and S1 Appendix). Such currents usually last several tens of ms after the peak of activation, and are considered brought about by the overlapping between activation and fast inactivation ("window current"), mainly evident in Na_V_1.9 isomer [42].

The experimental data here considered for Na_V_1.6 [28], showed that also this isomer exhibited a prominent persistent current. Yet, the current was only recorded immediately after obtaining the whole-cell voltage-clamp configuration, and became progressively reduced in subsequent recordings. The proposed model is able to reproduce such short-lived persistent current (Fig 7C) by suitably varying the parameters of I1 to C1 and O1 to I1 transitions.

### Implementation examples

This subsection is only intended as a not exhaustive proof of concept of the feasibility and suitability of the proposed channel model to be implemented in different types of computational models. As such, no in-depth exploration of the implementations here presented has been performed.

#### Comparison with an HH model

Although a detailed analysis of the HH formalism limitations are beyond the scope of the present paper, some modelling issues will be here considered.

**Fig 8:**
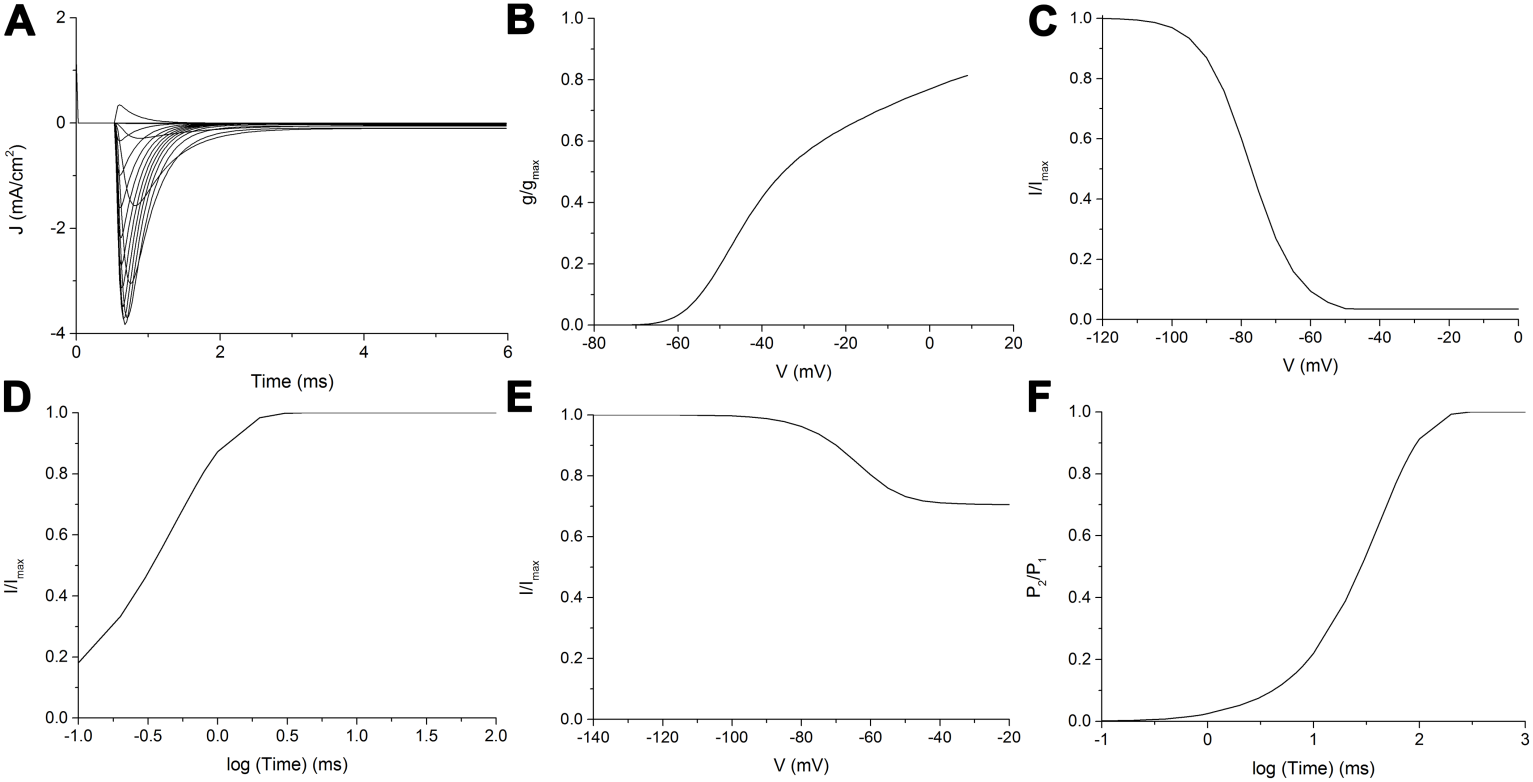
Electrophysiological features of a classical HH model of sodium channel. A: Voltage-clamp curves from -80 mV to 60 mV in step of 10 mV. B: Voltage dependence of the normalized conductance. C: Voltage dependence of normalized current during fast inactivation. D: Recovery from fast inactivation. E: Steady-state availability during slow inactivation. F: Recovery from slow inactivation.

Fig 8 shows how a previously published classical HH model of a sodium channel [38] behaves when examined by means of the electrophysiological protocols here considered.

The most striking features of the HH channel model by Dodge and Cooley [38] are the lack of a slow component for both entry into inactivation and repriming, and the very short time constant of repriming (0.49 ms, Fig 8D).

According to the HH formalism, the sodium current dependence on voltage is described by the well-known formula:

$$I_{Na}={\bar{g}}_{Na}\cdot m^{3}\cdot h\cdot \left(V-V_{Na}\right)$$ (21)

where *m* and *h* are, respectively, the activation and inactivation gating particles. They are assumed to undergo first-order transitions between nonpermissive and permissive forms, with rates satisfying the differential equations:

$$\frac{dm}{dt}=\alpha_{m}\left(1-m\right)-\beta_{m}m$$ (22)

$$\frac{dh}{dt}=\alpha_{h}\left(1-h\right)-\beta_{h}h$$ (23)

where *α* and *β* are the forward and backward voltage-dependent rate constants of transitions between nonpermissive and permissive forms.

To account for the slow inactivation by means of the HH formalism, a third gating particles is usually added [43], named *s*, whose kinetic behaviour is also described in terms of voltage-dependent rate constants, so that the Eq (21) is rewritten:

$$I_{Na}={\bar{g}}_{Na}\cdot m^{3}\cdot h\cdot s\cdot \left(V-V_{Na}\right)$$ (24)

By adding a third particle for slow inactivation to the original sodium channel model by Dodge and Cooley [38], it is possible to provide a second slower entry into inactivation (Fig 8E), yet the recovery from inactivation still goes on with a single time constant, which is the slower one (Fig 8F).

Moreover, another limitation concerns the tuning of the time constant of recovery from inactivation. By varying the parameters of the HH model, we were not able to tune the too short time constant of repriming without altering in an unrealistic way the steady-state availability during fast inactivation. From the modeller standpoint, indeed, the HH formalism does not provide enough parameters to be set in order to replicate the various electrophysiological behaviour of the channel. To set, indeed, the decay from activation, the steady-state availability during fast inactivation and the recovery from fast inactivation, the HH formalism only offers the parameters of forward and backward rate constant of inactivation (*h*).

The model developed by Dodge and Cooley is a reduced model of spinal motoneuron equipped with sodium, potassium and leakage conductances [38] and comprises of a soma, an equivalent dendrite, an initial segment, a myelinated segment, a node, and an extra unmyelinated segment. In order to sample the suitability and versatility of our model, we simply substituted the original HH sodium conductance with the proposed kinetic model tuned with our Na_V_1.6 parameters. The Na_V_1.6 sodium channel is typically expressed in spinal motoneurons [2], and the electrophysiological features it shows (S1 Appendix) are more similar than other isomers to those of the Dodge and Cooley model (Fig 8A–8D).

**Fig 9:**
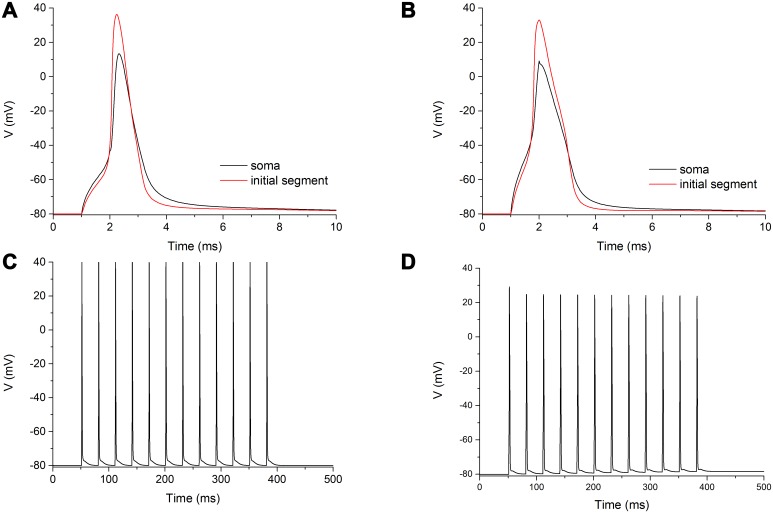
Comparison between an HH model and a ‘hybrid’ one. A: A single spike recorded at the soma (black trace) and initial segment (red trace) after a 75 nA, 1 ms long electrical stimulus at soma, in the neuron model by Dodge and Cooley [38]. B: A single spike recorded at the soma (black trace) and initial segment (red trace) after a 100 nA, 1 ms long electrical stimulus at soma, in the neuron model equipped with the Na_V_1.6 kinetic model. C: A series of spikes recorded at the soma following a repetitive stimulation in the HH model. D: Same recording and stimulating parameters as in C, in a ‘hybrid’ model. See text for details.

Apart from the substitution of the original sodium conductance with our Na_V_1.6 model, we only modified the conductance densities (or maximal conductances) at the initial segment for sodium (from 0.6 to 1.2 S/cm^2^) and potassium (from 0.1 to 0.07 S/cm^2^) channels, and set the resting potential to -80 mV, instead of the old convention of 0 mV (in turn also consequently moving to more hyperpolarized values the hemiactivation and heminactivation parameters of the classical model). By mantaining unaltered all other biophysical and structural parameters, our ‘hybrid’ neuron model exhibited, when stimulated by a virtual electrode located into the soma, a typical spike (Fig 9B), similar to the original one (Fig 9A). The only discernible differences were a slightly wider duration of the spike and an increased threshold of the stimulus (~65 nA *versus* ~85 nA) to evoke the spike.

In order to compare the computational load between the two models, a 500 ms long simulation was performed, in which a train of electrical stimuli (300 ms long, with an amplitude of 100 nA and a frequency of 33 Hz) was delivered to the soma (Fig 9C and 9D). According to the expectations, due to the minor computational load of the HH formalism, the original model completed the simulation in 0.44 s, while the ‘hybrid’ one ended the run in 1.43 s (time step of integration: 0.025 ms).

The Supporting Information file (S2 Appendix. Examples of model channel implementation) provides two other examples of the implementation of the proposed channel model. They show a) how the model is computationally more efficient than previously developed more complex kinetic models, and b) how it behaves in morphologically detailed neuron models.

## Discussion

In this study, we developed a single unifying deterministic Markov-type kinetic model which consistently accounts for the macroscopic electrophysiological behaviour of all human VGSC isomers, Na_V_1.1 to Na_V_1.9.

To date, the developed general model appears to be the simplest and most parsimonious one, able to account in detail for a number of electrophysiological features derived from experimental data, and suitable to be implemented in biologically detailed, conductance-based neurons and neural networks models.

The kinetic model here proposed bridges the gap between what is available from more recent electrophysiological studies and what is needed to construct reliable VGSCs models for use when simulating neural activity [20, 44]. In addition, its implementation in different types of neuron models has been proven straightforward and suitable.

Moreover, though the model is targeted to human VGSCs, its versatility makes it easily of use to simulate VGSCs from other species, provided that experimental data are available to constrain its parameters.

In modelling studies the level of approximation to the physical reality can be made strictly dependent on the temporal and spatial scale and on the complexity of the investigated issue. Thus, when dealing with the macroscopic scale of multiple interacting neural networks, simplified neurons are usually adopted [45–46], without even considering the types and amount of ionic channels which in real neurons are responsible of the electrical behaviour of the cell. On the other hand, in ion-channel modelling, the microscopic biophysical detail can be moved forward to account for the minimal displacements of the sensory domain of the pore-forming protein (e.g., [47]) in response to a transmembrane voltage change. Yet, the biophysical detail also brings a considerable computational load which limits the use of these detailed ion-channels models in simulations of neurons and large networks of those. In this study, in accordance with a common practice within computational neuroscience, a third intermediate way has been adopted, focused on the mesoscopic scale of the electrophysiological behavior of the VGSC. This modelling study, indeed, exclusively deals with the macroscopic ionic currents of the VGSC, which are of interest when building biophysically detailed neurons and neural network models, rather than their discrete and stochastic counterparts at the molecular level.

### Markov-type models

In their pioneering and seminal works, Hodgkin and Huxley combined the voltage-clamp techniques and quantitative modelling to provide a deterministic and continuous description of the macroscopic ionic currents [3]. They were able to clarify the nonlinear behavior of ions permeation through the cellular membrane in response to membrane depolarizations, and to disclose the relationship between these ions fluxes and the axonal spike. As a consequence, a wealth of data about the electrophysiological behaviour of the excitable membranes were provided with a fairly accurate mathematical description: from the form, amplitude and velocity of a propagated action potential to the subthreshold depolarizations, to the refractory period after a spike, to the inward sodium and outward potassium movements associated to an impulse.

However, thanks to the patch-clamp techniques [13], and the discovery of the molecular structure of the pore-forming proteins [14], it became clear that excitable membranes are studded with discrete ion channels undergoing random fluctuations between open and closed stable states [8]. In this respect, the HH formalism appeared as a simple macroscopic and deterministic description of a phenomenon that ultimately arises from the microscopic and stochastic behaviour of the system [9].

Markov-type models have been proposed as efficient kinetics scheme, suitable to capture the essential properties of a number of neural structures, like voltage-gated channels, transmitter-gated channels and second messenger-activated channels [40].

A Markov model represents an ion channel as a collection of states and a set of transition probabilities between them, and rely on the assumptions that: a) the configuration of a channel protein can be operationally grouped into a set of distinct states separated by large energy barriers, and b) the probability of state transitions is dependent only on the present state occupied [1, 40].

The various states represent a sequence of protein conformations that underlies the gating of the channel. The time evolution of the probability of state *S*_*i*_ is described by the *Master equation* [48]:

$$\frac{dP\left(S_{i},t\right)}{dt}=\sum_{j=1}^{n}P\left(S_{j},t\right)P(S_{j}\to S_{i})-\sum_{i=1}^{n}P\left(S_{i},t\right)P\left(S_{i}\to S_{j}\right)$$ (25)

where *P(S*_*i*_, *t)* is the probability of being in a state *S*_*i*_ at the time *t*, and *P(S*_*i*_*→S*_*j*_*)* is the transition probability from state *S*_*i*_ to state *S*_*j*_.

In the limit of large numbers of identical channels, the quantities given in the master equation can be reinterpreted. The probability of being in a state *S*_*i*_ become the fraction of channels in state *S*_*i*_, noted *s*_*i*_, and the transition probabilities from state *S*_*i*_ to state *S*_*j*_ becomes the rate constants, *r*_*ij*_, of the reactions

$$\begin{array}{c} r_{ij}\\ S_{i}⇄S_{j}\\ r_{ji} \end{array}.$$ (26)

In this case, the master equation can be rewritten as:

$$\frac{ds_{i}}{dt}=\sum_{j=1}^{n}s_{j}r_{ji}-\sum_{i=1}^{n}s_{i}r_{ij}$$ (27)

which is a conventional kinetic equation for the various states of the system [49].

Stochastic Markov models, as in Eq (25), are adequate to describe the stochastic behaviour of ion channels as recorded using single-channel recording techniques [50]. In other cases, where a large number of ion channels are involved, as in the whole-cell patch-clamp recordings here considered, the macroscopic currents are continuous and more adequately described by conventional kinetic equations, as in Eq (27).

In the former case, derived from single-channel recordings, a Markov model is usually designated starting from ion-channel molecular representation, with each state of the model corresponding to a different configuration of the molecule. This approach gives a better understanding of the biophysical structure and functioning of the channel, and, by taking into account also the smaller gating currents, it details even the minimal, non-conducting molecular displacement.

Stochastic Markov models derived from single-channel recordings in ligand-gated ion channels have proven to be able to solve the inverse problem, that is the direct fitting of the models with raw data, with provision of estimates for rate constants and estimation of the errors for those estimates [51–52].

However, it is also possible to take a signal-processing approach to the design of Markov models, [20, 49, 53]: the required model is the minimal model that represents with sufficient accuracy the response of the channel to the stimulation protocols. This approach leads to more economical models, more suitable for numerical simulations of large collections of channels and of neurons, and was followed in the present paper.

In particular, this phenomenological approach is more reminiscent of the empirical and classical fitting of ionic macroscopic currents developed by Hodgkin and Huxley [3]. It could be argued that such approach is more similar to the fitting of a curve and hardly suitable to reveal the finest details of the biophysical features of the channel.

Yet, this is specifically in agreement with the main goal of the present study, which was to develop a model without the structural HH limitations and able to include the most recent experimental data on the macroscopic currents of VGSCs.

The phenomenological approach is intended to develop the smallest number of states and transitions necessary to replicate the electrophysiological VGSCs behaviour. Consequently, there is no exact correspondence of the model states with the physical states of the channel. In other words, in our phenomenological model the states do not represent single physical events, but each of them should be considered as an aggregate of molecular configurations suitable to be treated as a functional entity. For example, while a series of four (or more) closed states are commonly hypothesized (usually in adherence with the tetrameric structure of the proteic channel) before an open state can develop following a depolarizing step, our model collapses them all in only two. Two closed states, indeed, are necessary and sufficient to deterministically reproduce: a) the tail currents after a brisk repolaritation, b) the kinetics of the activation sequence, c) the kinetics of fast inactivation.

The aim to develop the computationally lightest model allows us to make one transition practically irreversible (from O1 to I1), and releases the phenomenological model from the principle of microscopic reversibility, like other kinetic schemes based on macroscopic currents (see [54], for a recent example). Microscopic reversibility, indeed, only holds when the states are elementary processes (collisions, molecules, elementary reactions, etc). On the other hand, most, but not all, ion channels obey the law of microscopic reversibility [55], and the law is only true at genuine equilibrium, which could not be the case when some sort of external energy supply is involved (in this case, an ionic gradient) [55].

### Previous examples of sodium channel kinetic models

Kuo and Bean [41] proposed a Markov-type model of VGSC incorporating the results of their study on the kinetics of the recovery from inactivation of sodium channel in rat hippocampal CA1 neurons. Since then, the model or its variants have been adopted as a more detailed alternative compared to the HH models [11, 56–59]. Yet, being provided with 12 states and 32 transitions, its computational load makes it quite heavy to be implemented in multi-compartmental neuron models and networks of those.

Mimicking the open probability of the HH model, an 8-state Markov-type kinetic model of voltage-gated ion channels has also been proposed [59]. More recently, aimed at modelling the slow inactivation of VGSC, a new version of the model has been proposed [27] for the isomer Na_V_1.5, derived from the 8-state model by Milescu et al [59]. To account for the slow inactivation, 4 inactivated states were added, and all the transitions between states (similarly to the present model) were made voltage-dependent. The resulting model (Figure 3B in [27]) fits very smoothly the complex electrophysiological behaviour of Na_V_1.5, yet it is equipped with 12 states and 34 transitions.

Compared to the model by Zhang [27], the one here proposed is able to simulate in detail the phenomenological behavior of Na_V_1.5 as well as of each other VGSC isomers with a significantly lower number of states and transitions (6 and 12, respectively).

### The need for detailed kinetic models of ionic channels

In recent years, the increasing availability to the scientific community of powerful computational systems [44, 60] able to process, even in parallel, huge amount of data in relatively short time, has promoted the development of simulations of complex neural structures [4–7], equipped with large amounts of neural cells and synaptic connections. These simulations tried to be realistic and biologically inspired as much as possible, according to a bottom-up plan, and they succeeded, indeed, in replicating a number of electrophysiological experimental features of the cells.

In such bottom-up approaches, however, the value of detailed kinetics of ionic channels, as building blocks of cells electrophysiology, cannot be ignored. Taking examples from the VGSC here described, the presence of complex kinetics with fast and slow inactivations must be considered, as they have direct effects on the recovery from inactivation and, consequently, on the refractory time of the cells which, in turn, affects the ability of the cell to fire repetitive action potentials.

In addition, it should be considered that biologically inspired models have to consistently deal with the diversity and variety of the different channel isoforms. A differential topographic clustering of distinct ionic channels isomers, indeed, has been described also in subcellular compartments [61]. In real neurons, indeed, also subtle differences in ion-channels kinetics do have relevance. This is mainly showed with the clearest evidence by the effects of genetic mutations affecting ion-channels genes, considered pathogenic in a number of channelopathies. As regard to the VGSC, indeed, even slight differences in kinetics sustained by the mutation can give rise to severe diseases [21–22, 30].

### Limitations and future research

The present work is a purely modelling study, and the proposed model relies on previously published experimental data. On one hand, this limits the genuine interpretation the modeller can actively draw from the raw experimental data. On the other hand, it is probably not affordable for a single laboratory to conduct research on the electrophysiological behavior of all the VGSC isomers, and data have necessarily to be collected from different studies.

Lack of interaction with experimenters also acts in the opposite way, as some suggestion of not canonical experimental paradigm (e.g. the adoption of different levels of repolarization during inactivation protocols), arisen during modelling studies, cannot be performed.

Moreover, different laboratories often perform experiments with not exactly similar protocols, and in some cases not all the useful experimental data are available. One example is the slow component of the recovery from inactivation (Table 1), where the second slower time constant of recovery can be only discernible by electrophysiological protocols carrying a long conditioning pulse, followed by longer repolarizing intervals, before the test pulse. A further example is the two time constants of fast inactivation or the ultra-slow inactivation, which have been quantitatively reported on in only few studies.

A further limitation is that some less investigated VGSC electrophysiological features, like the resurgent currents, have not been accounted for in this study.

An ensuing follow-up of the present study will be to evaluate the suitability of the proposed model as a general kinetic model for all voltage-gated ionic channels with similar molecular structure (four-metameric pore-forming proteins with six transmembrane domains in each metamere), calcium and potassium ion channels *in primis*.

## Supporting information

S1 Appendix Modelled graphics with reference to the original ones. (DOCX)

S2 Appendix Examples of model channel implementation. (DOCX)

S1 Fig Electrophysiological features of a 14-state kinetic model of a fast sodium channel. A: Voltage-clamp curves from -80 mV to 60 mV in step of 10 mV. B: Voltage dependence of the normalized conductance. C: Voltage dependence of normalized current during fast inctivation. D: Recovery from fast inactivation.
(TIF)

S2 Fig Comparison with a 14-state kinetic model of sodium channel. A: Baseline autonomous spiking in a reduced neuron model of cholinergic striatal interneuron. B: After substitution of the original kinetic fast VGSC with the Na_V_1.2 model proposed, the model is able to reproduce the pacemaker discharge as well. C: Baseline activity in neuron model deprived by fast VGSC, all other parameters unchanged. D: Single action potential from the spiking train of the original model [1]. E: Single action potential after substituting the original fast sodium kinetic channel with the Na_V_1.2 VGSC of our model.
(TIF)

S3 Fig Implementation in a morphologically detailed neuron model. A: Digitized detailed 3D somato-dendritic morphology of a spinal motoneuron imported from NeuroMorpho.org [4] and implemented in a computational model [3]. B: Action potential evoked in the original model by an electrical impulse delivered at the soma. C: A similar spike obtained after substituting the original HH sodium channels with the Na_V_1.2 and Na_V_1.6 kinetic models.
(TIF)
