# Supplementary material for: A single Markov-type kinetic model accounting for the macroscopic currents of all human voltage-gated sodium channel isoforms
Source: PLoS Comput Biol. 2017 Sep 1;13(9):e1005737. doi: 10.1371/journal.pcbi.1005737 (PMC5599066; doi:10.1371/journal.pcbi.1005737)
Supplement: S1 Appendix — (DOCX) [file pcbi.1005737.s001.docx]

**Sodium channels**

**Nav1.1**

Simulation data

Voltage-clamp: -80 mV to 70 mV in step of 10 mV (for experimental data see Rhodes et al, 2004; Fig 1A. Simulation data for wild-type variant, WT-SCN1A)


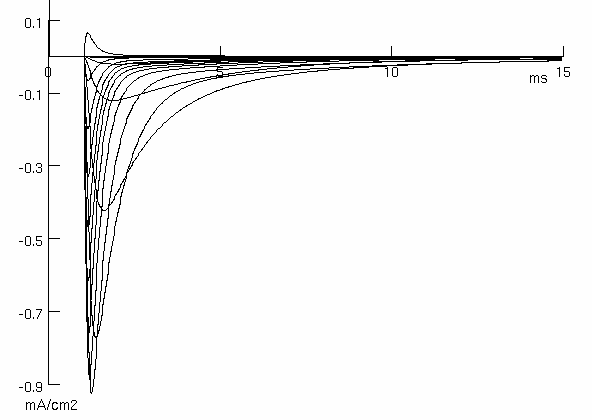


Voltage-current relationship (see Rhodes et al, 2004; Fig 3A)


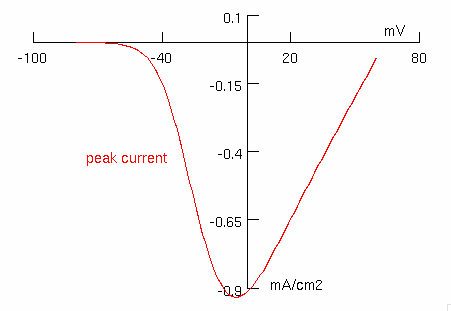


Voltage dependence of the normalised conductance (see Rhodes et al, 2004; Fig 3B)


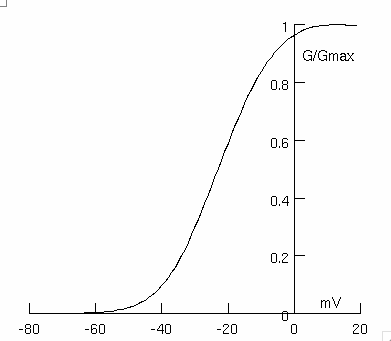


Voltage dependence of normalized current during fast inactivation (see Rhodes et al, 2004; Fig 3C)


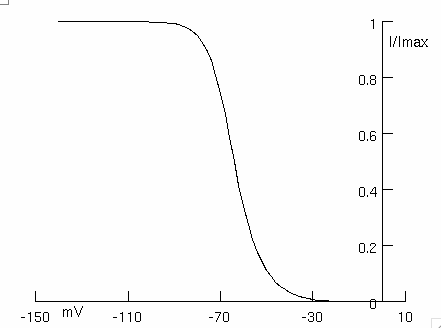


Recovery from fast inactivation (see Rhodes et al, 2004; Fig 3D)


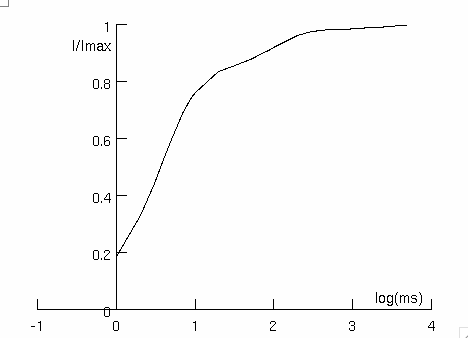


Time constants of inactivation (see Rhodes et al, 2004; Fig 2B)


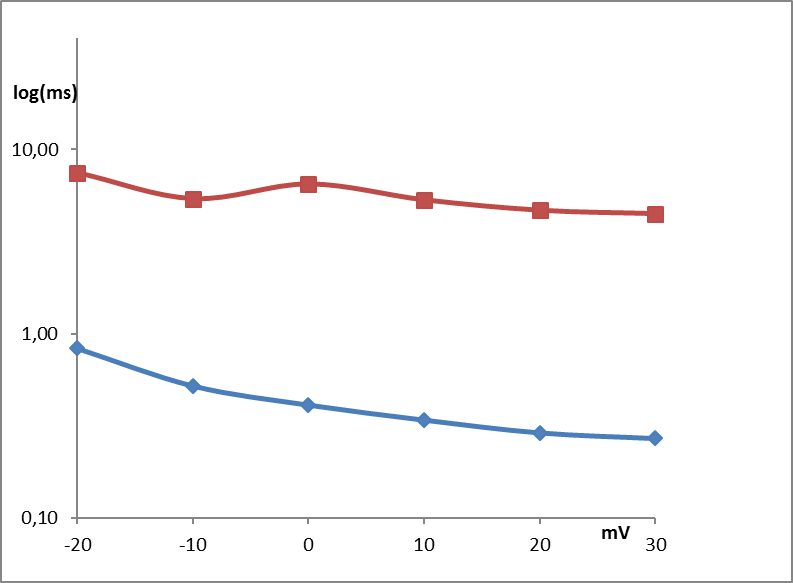


Onset of slow inactivation (see Rhodes et al, 2004; Fig 5A)


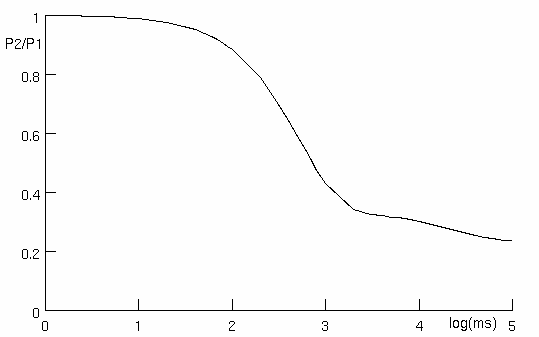


Steady-state slow inactivation (see Rhodes et al, 2004; Fig 5B)


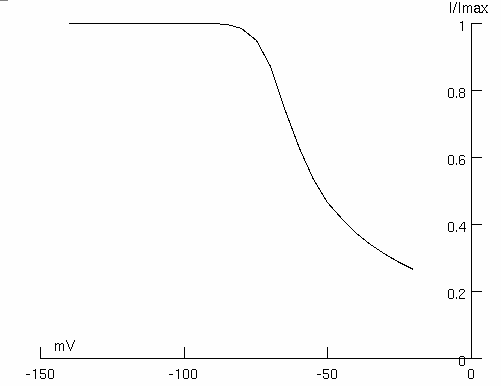


Slow inactivation recovery (see Rhodes et al, 2004; Fig 5C)


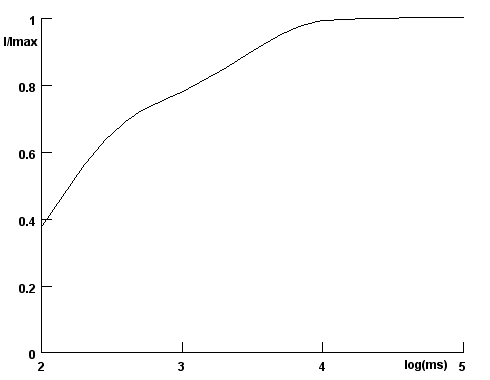


**Nav1.2**

Simulation data

Voltage-clamp: -80 mV to 70 mV in step of 10 mV (see Misra et al, 2008; Fig 1B. Simulation data for wild-type variant, WT-SCN2A)


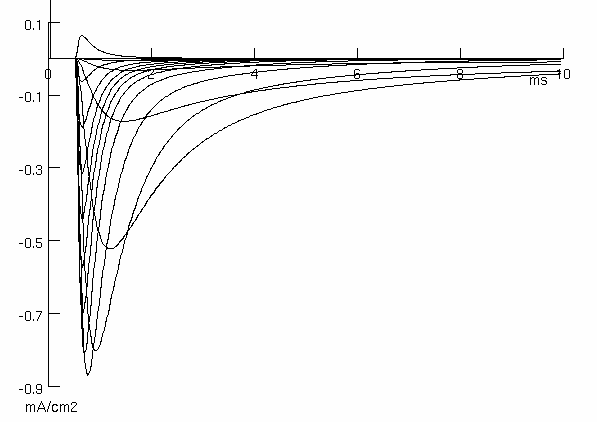


Voltage-current relationship (see Misra et al, 2008; Fig 2A)


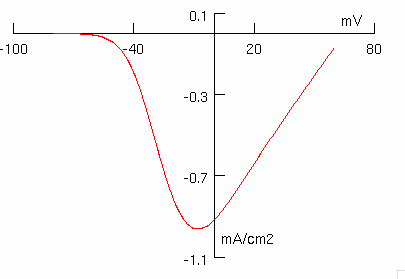


Voltage dependence of the normalised conductance (see Misra et al, 2008; Fig 2B)


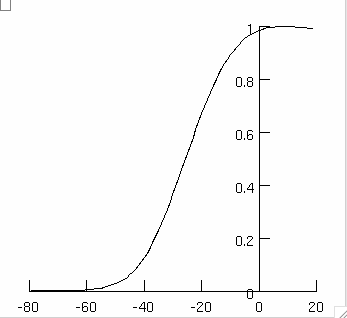


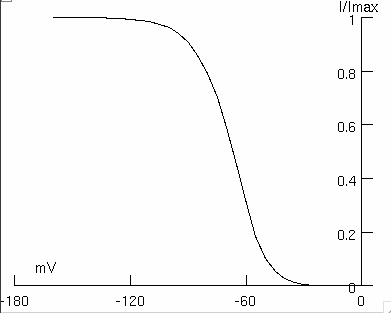
Voltage dependence of normalized current after steady-state fast inactivation (see Misra et al, 2008; Fig 2C)

Recovery from fast inactivation (see Misra et al, 2008; Fig 3D)


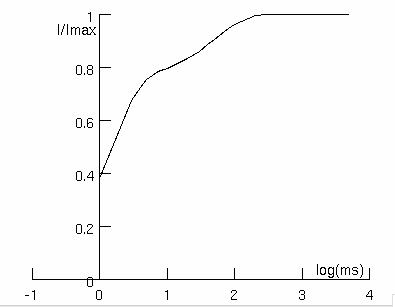


Time constants of inactivation (see Misra et al, 2008; Fig 3A)


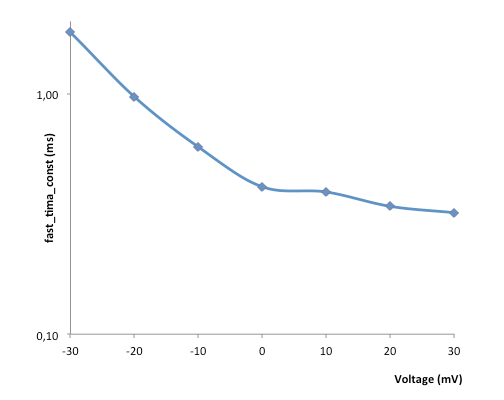


**Nav1.3**

Simulation data

Voltage-clamp: -90 mV to +40 mV in step of 10 mV (see Cusdin et al, 2010; Fig 1A: Simulation data for Na_V_1.3/no β3)


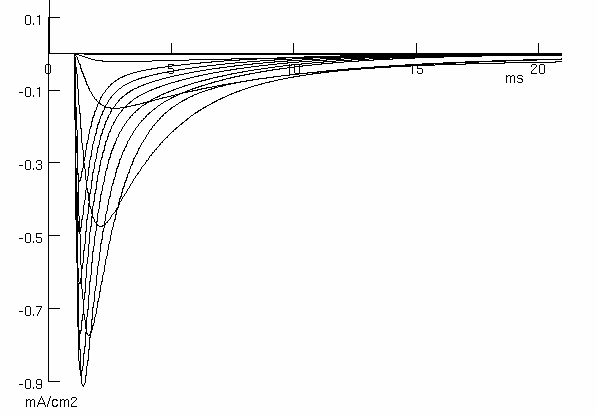


Voltage dependence of the normalised conductance (see Cusdin et al, 2010; Fig 1B)


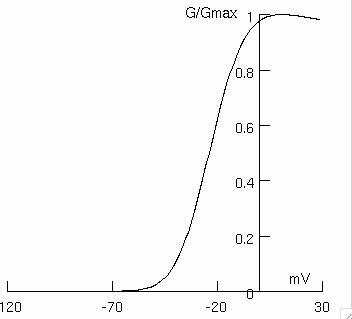


Voltage dependence of normalized current after steady-state fast inactivation (see Cusdin et al, 2010; Fig 2B)


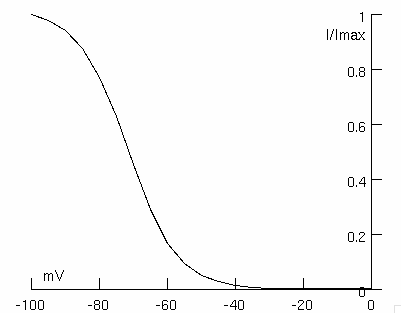


**Nav1.4**

Simulation data

Voltage-current relationship (see Arnold et al, 2015; Fig 4A. Simulation data for wild-type variant)


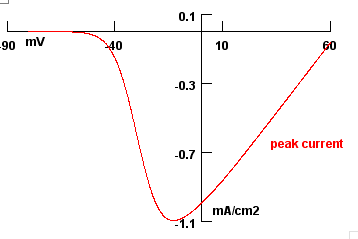


Voltage dependence of the normalised conductance (see Arnold et al, 2015; Fig 4B)


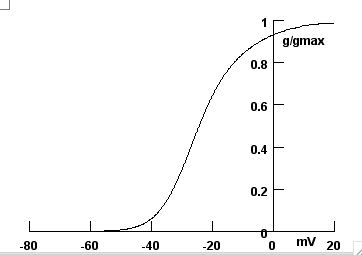


Steady-state availability during fast inactivation (see Arnold et al, 2015; Fig 4C)


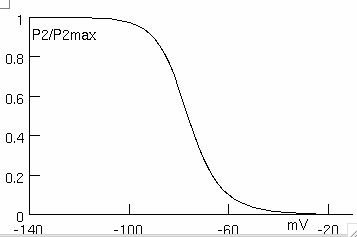


Recovery from fast inactivation (see Arnold et al, 2015; Fig 4D)


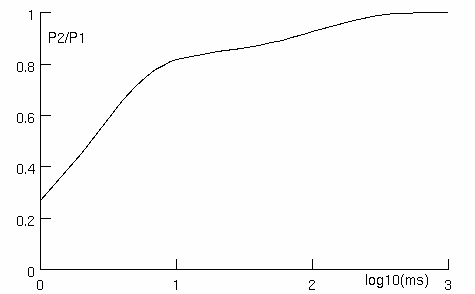


Fig 4E

Time constant of inactivation (see Arnold et al, 2015; Fig 4E)

**
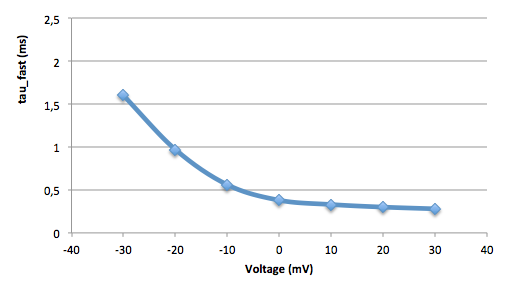
**

Onset of slow inactivation (see Arnold et al, 2015; Fig 6A)

**
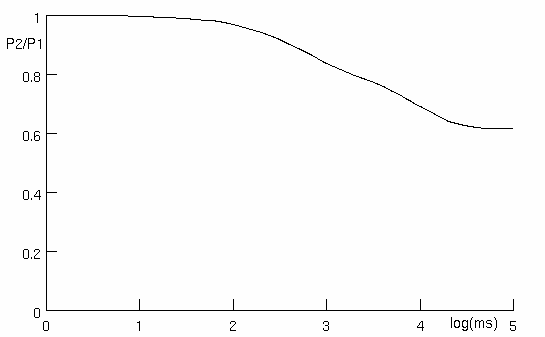
**

Steady-state slow inactivation (see Arnold et al, 2015; Fig 6B)


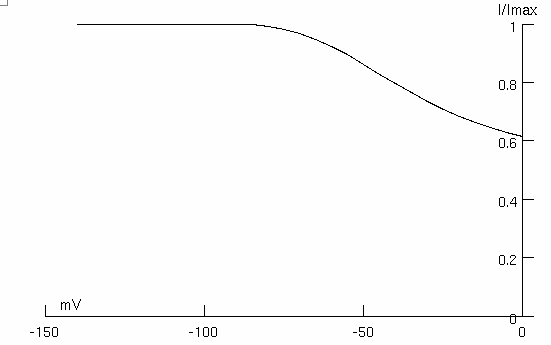


Slow inactivation recovery (see Arnold et al, 2015; Fig 6C)


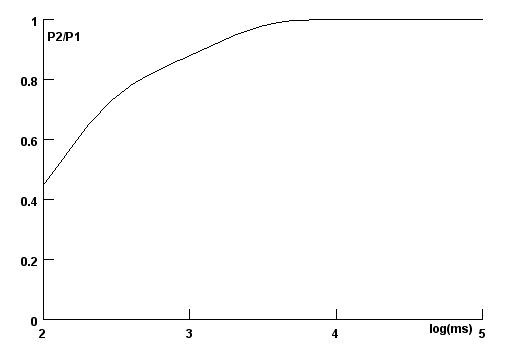


**Nav1.5**

Simulation data

Voltage-clamp: -90 mV to 60 mV in steps of 5 mV (see Zhang et al, 2013; Fig 1A)

**
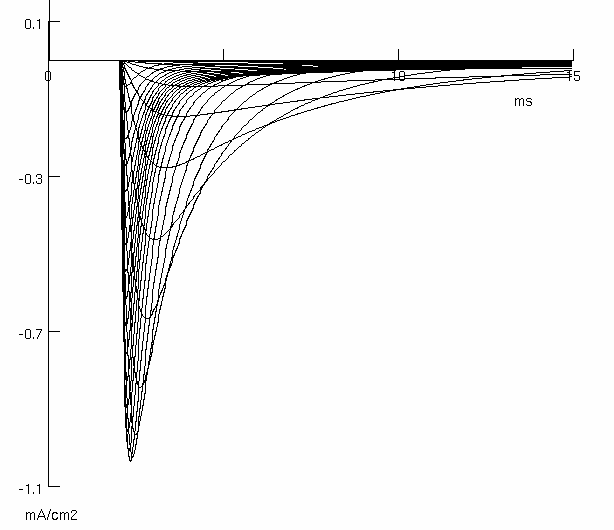
**

Fig 1D

Voltage dependence of the normalised conductance(see Zhang et al, 2013; Fig 1D)


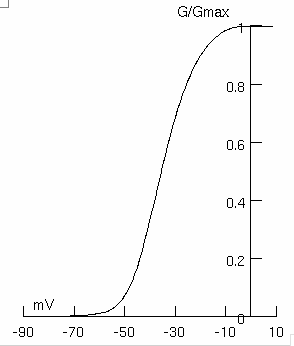


Steady-state availability during fast inactivation (see Zhang et al, 2013; Fig 1E)


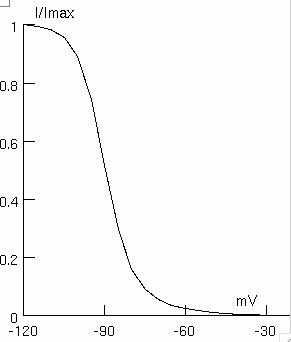


Recovery from fast and slow inactivation (see Zhang et al, 2013; Fig 2D)


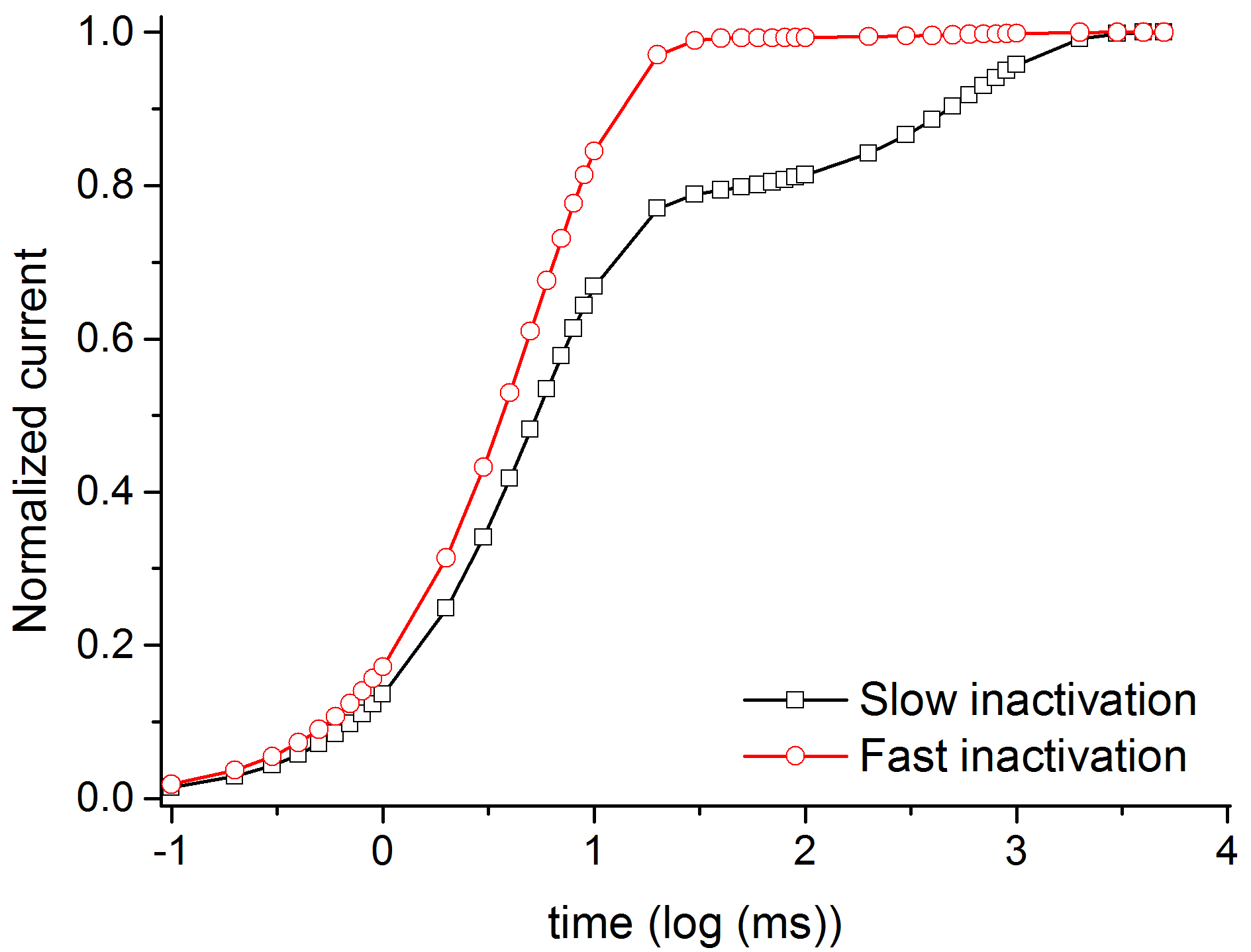


Development of slow inactivation (see Zhang et al, 2013; Fig 2F)


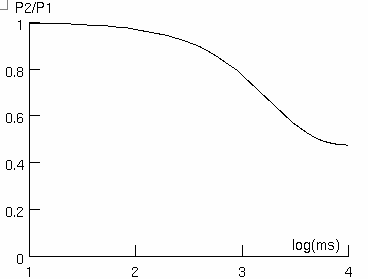


Availability curves (see Zhang et al, 2013; Fig 1B)


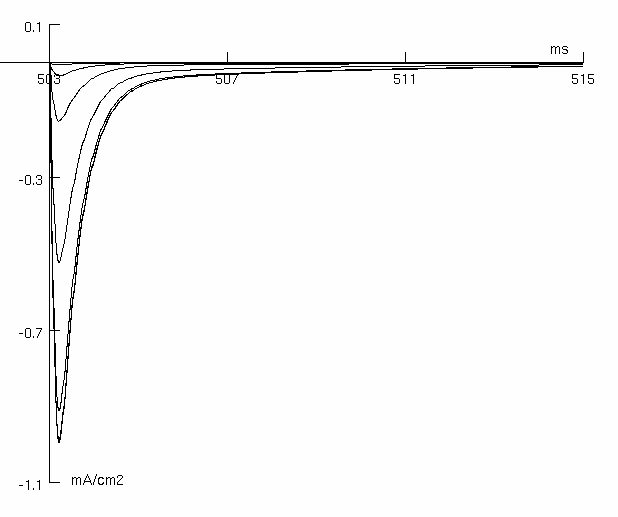


Deactivation curves (see Zhang et al, 2013; Fig 1C)


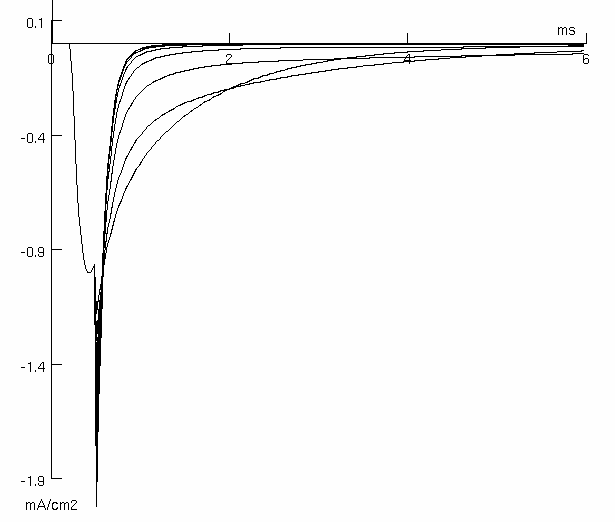


Fractional recovery of fast inactivation (repolarization voltages: -120, -110, -100, -90 mV) (see Zhang et al, 2013; Fig 2A)


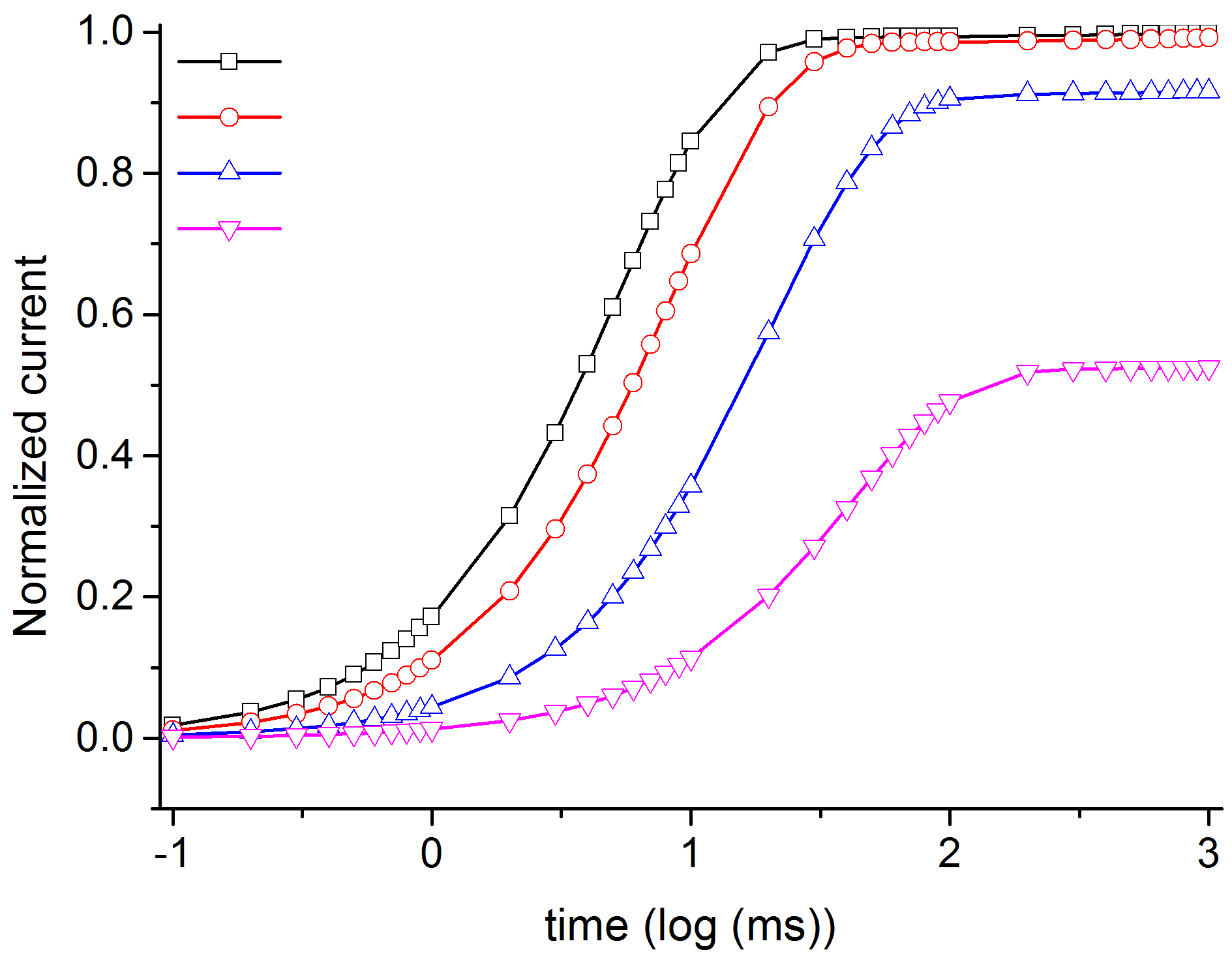


Inactivation time constant dependence from voltage (see Zhang et al, 2013; Fig 4E)


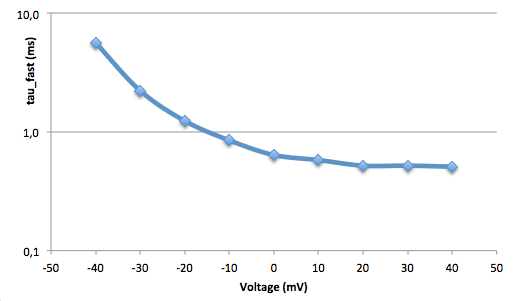


Deactivation time constant dependence from voltage (see Zhang et al, 2013; Fig 4F)


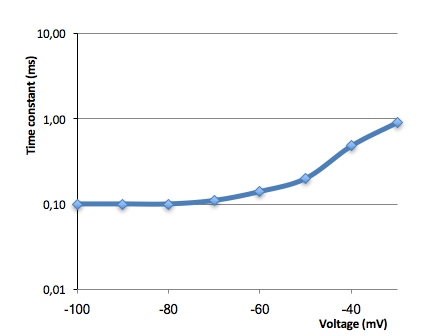


**Nav1.6**

Simulation data

Voltage-clamp: -70 mV to 70 mV in step of 10 mV (see Burbidge et al, 2002; Fig 2A)


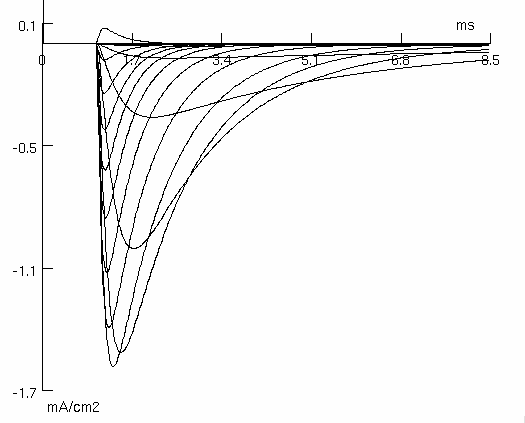


Fig 2C

Voltage-current relationship (see Burbidge et al, 2002; Fig 2C)


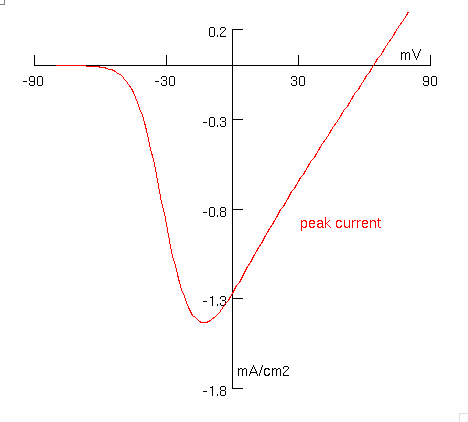


Voltage dependence of the normalised conductance (see Burbidge et al, 2002; Fig 2D)


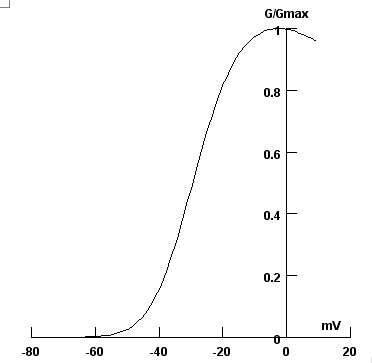


Voltage dependence of normalized current during both slow and fast inactivation (see Burbidge et al, 2002; Fig 3B)


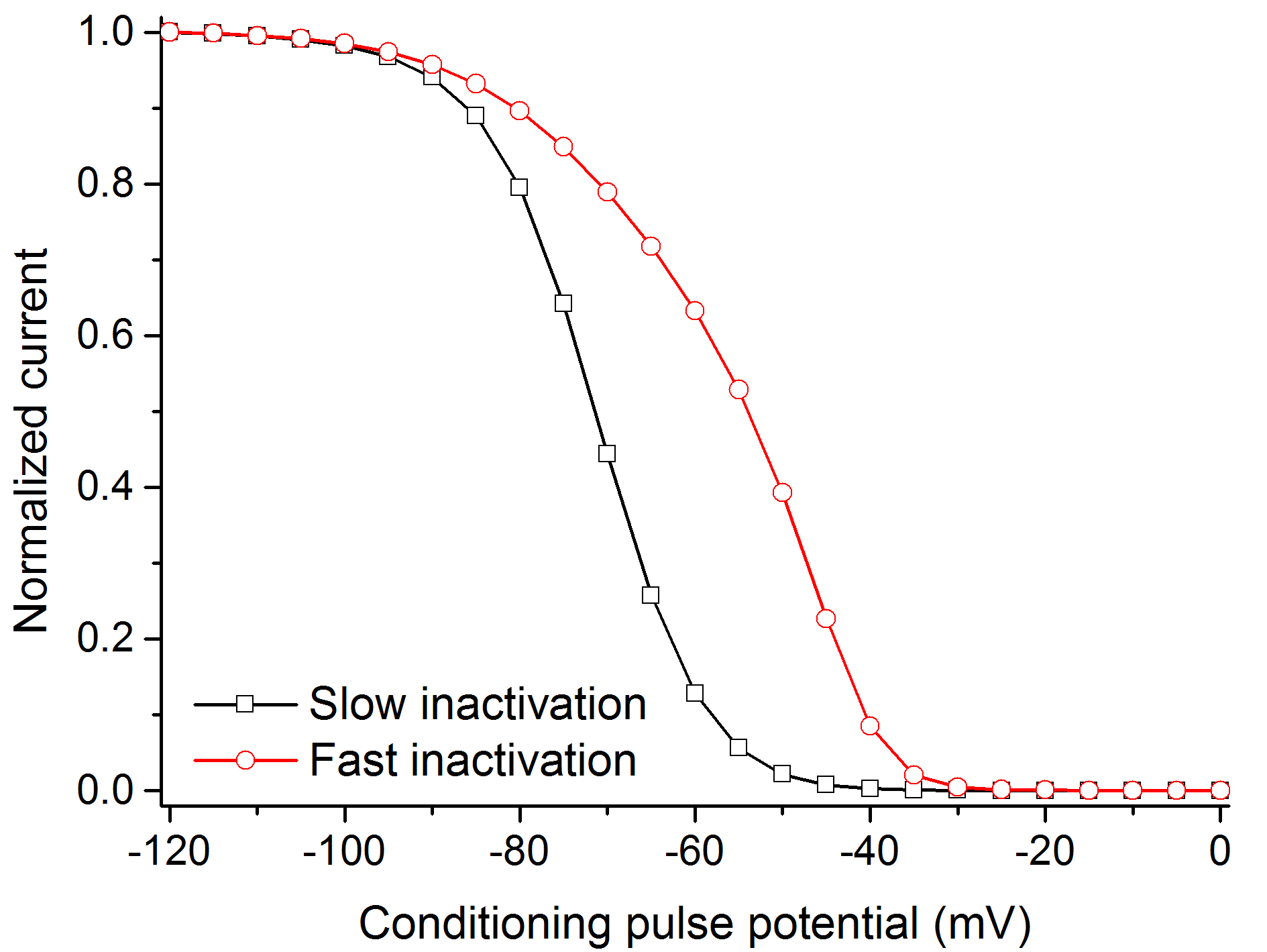


Recovery from inactivation (see Burbidge et al, 2002; Fig 3D)


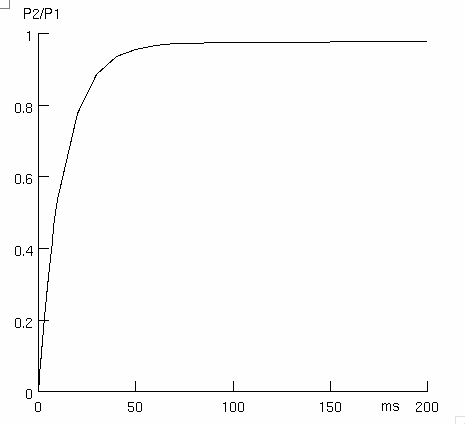


Time constant of inactivation (see Burbidge et al, 2002; Fig 3C)


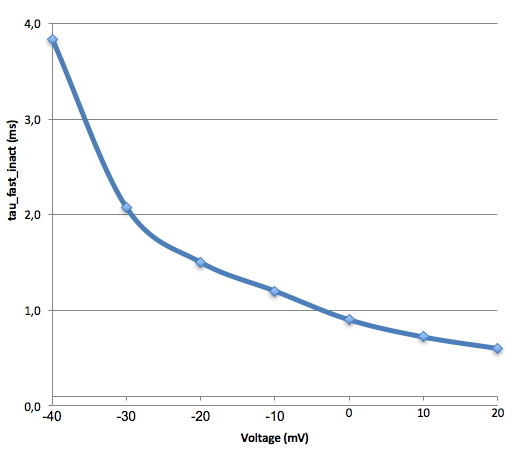


I-V curves with persistent current (see Burbidge et al, 2002; Fig 4A)


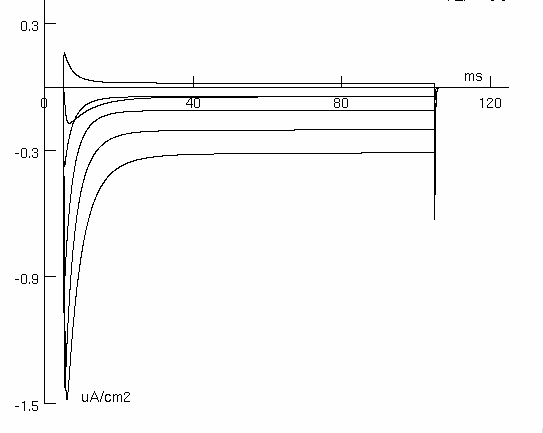


I-V relationship (see Burbidge et al, 2002; Fig 4C)


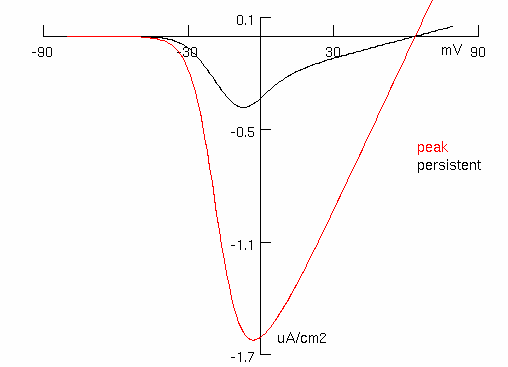


**Nav1.7**

Simulation data

Voltage-clamp: -80 mV to 70 mV in step of 10 mV (see Chatelier et al, 2008; Fig 1C. Simulation data for 5N11S variant))


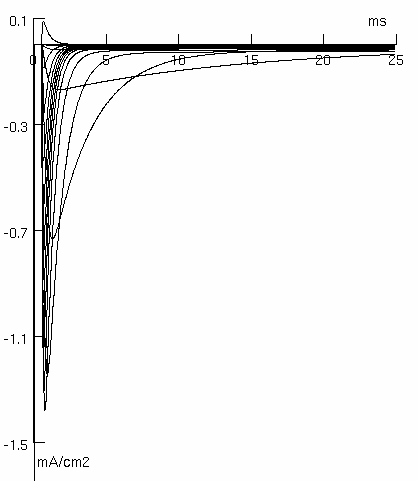


Voltage dependence of the normalised conductance (see Chatelier et al, 2008; Fig 2B)


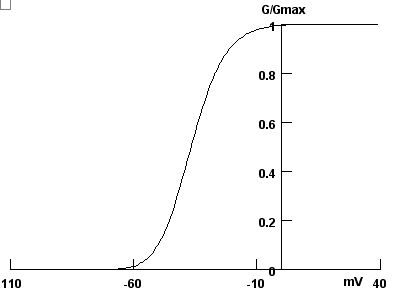


Fig. 2C

Voltage dependence of normalized current during fast inactivation (see Chatelier et al, 2008; Fig 2C)


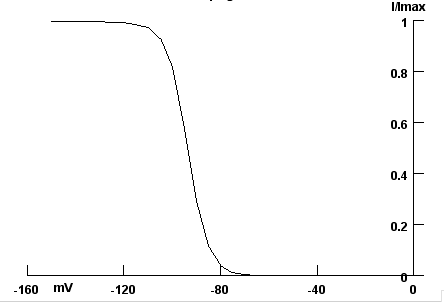


Recovery from inactivation (see Chatelier et al, 2008; Fig 4A)


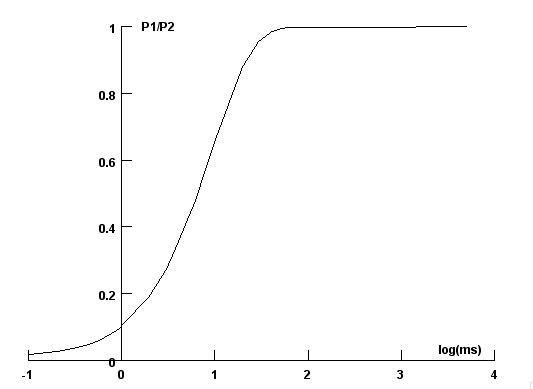


I-V relationship (see Chatelier et al, 2008; Fig 2A)


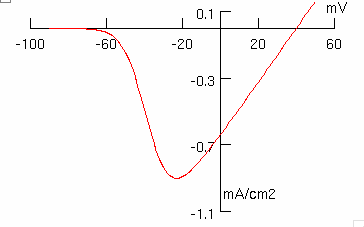


Time constant of inactivation (see Chatelier et al, 2008; Fig 3A)


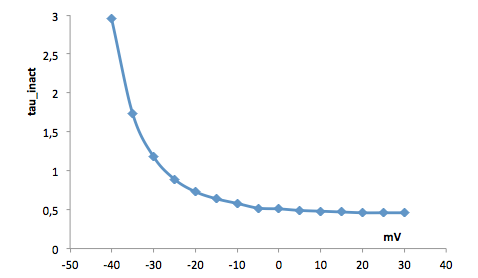


Time constant of deactivation (see Chatelier et al, 2008; Fig 3B)


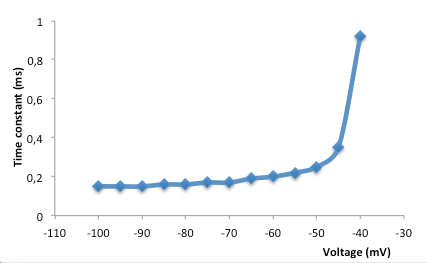


**Nav1.8**

Simulation data

Voltage-clamp: -80 mV to 55 mV in step of 5 mV (see Huang et al, 2013; Fig 1A)


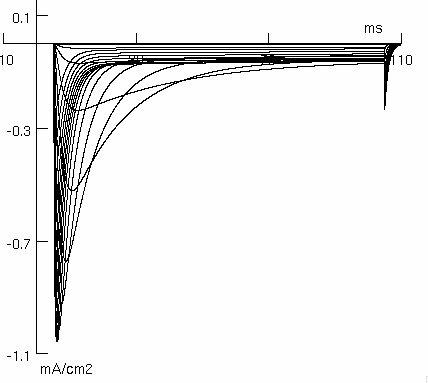


I-V relationship (see Huang et al, 2013; Fig 1C)


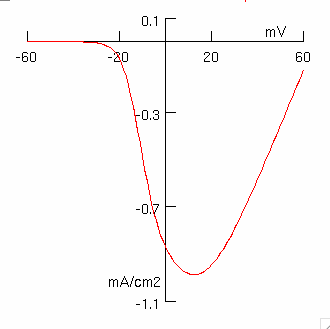


Voltage dependence of the normalised conductance and of the normalized current during inactivation

(see Huang et al, 2013; Fig 1D)


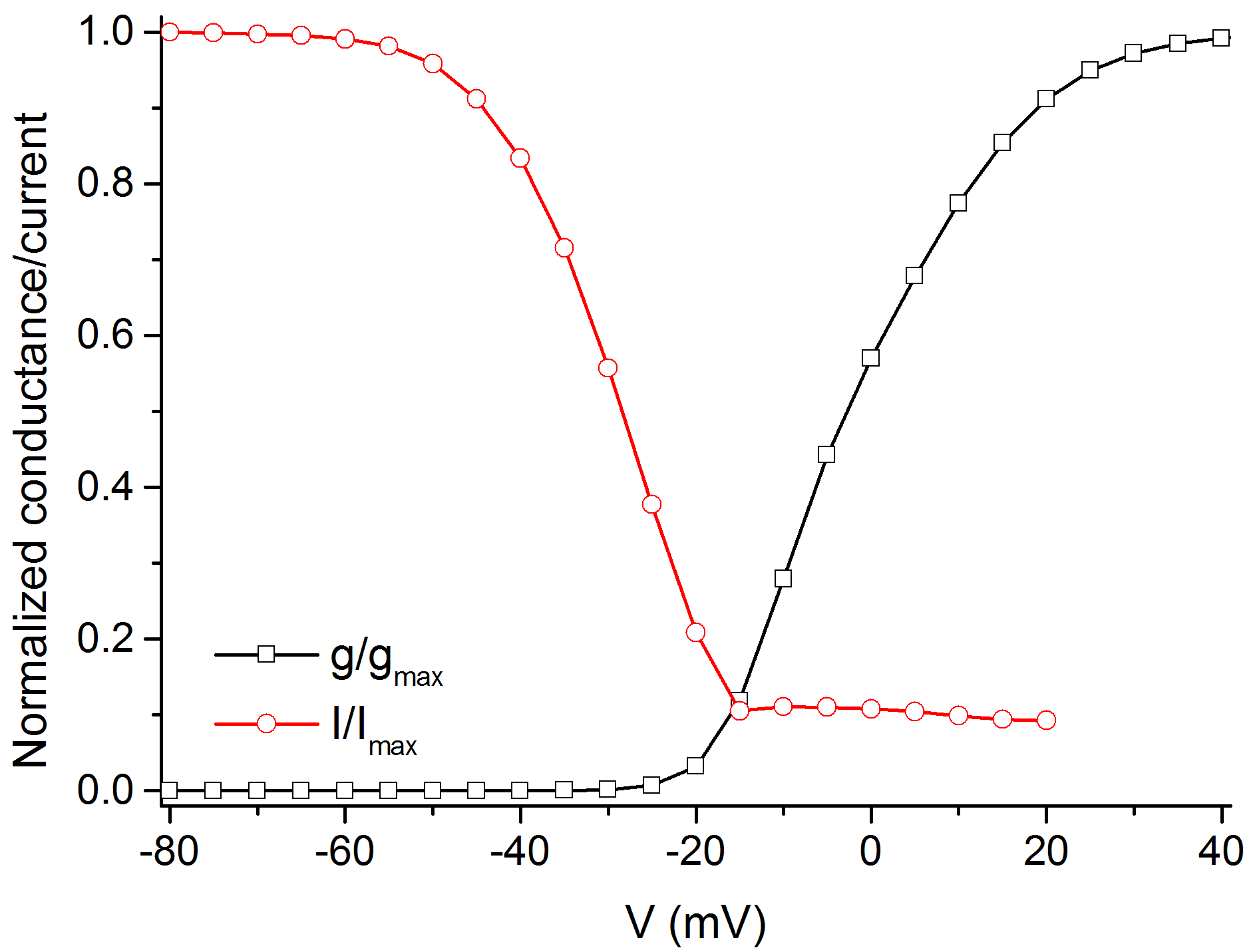


Recovery from inactivation (see Huang et al, 2013; Fig 3A)


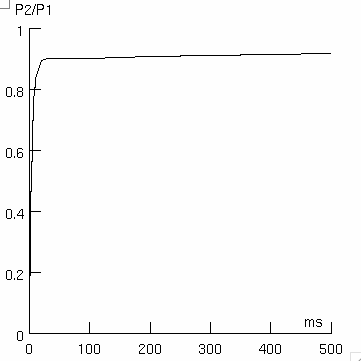


Inactivation time constant dependence from voltage (see Huang et al, 2013; Fig 2F)


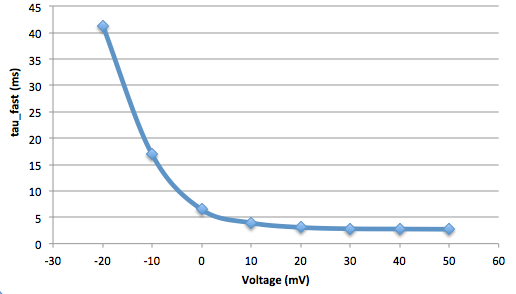


**Nav1.9**

Simulated data

Voltage-clamp: -70 mV to 50 mV in step of 10 mV (see Vanoye et al, 2013; Fig 1A)


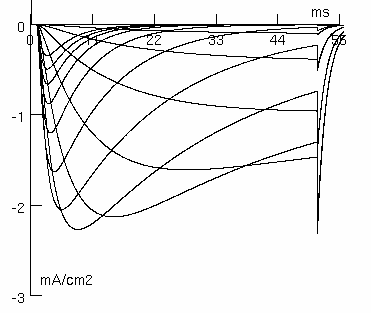


Normalized conductance-voltage relationship (see Vanoye et al, 2013; Fig 2F)


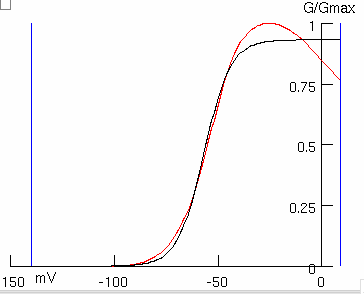


Voltage-current relationship (see Vanoye et al, 2013; Fig 1B)


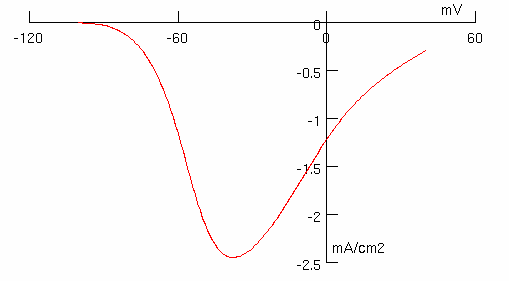


Voltage dependence of normalized current during inactivation (see Vanoye et al, 2013; Fig 2E)


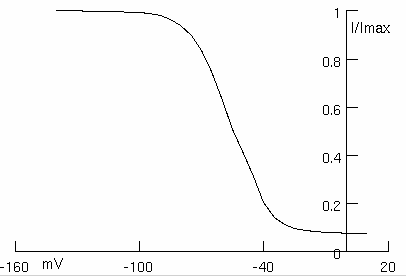


Recovery from inactivation (see Vanoye et al, 2013; Fig 2F)


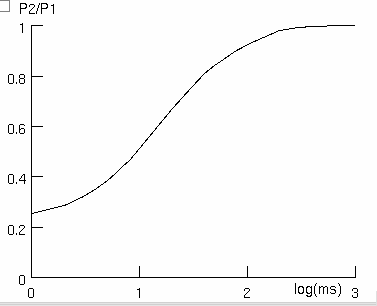


Inactivation time constant dependence from voltage (see Vanoye et al, 2013; Fig 2F)


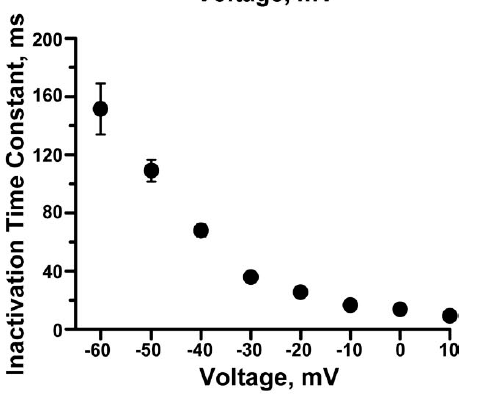


**
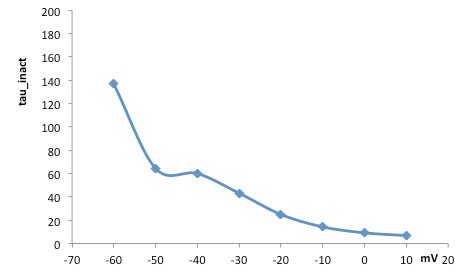
**

References

Arnold WD, Feldman DH, Ramirez S, He L, Kassar D, Quick A, Klassen TL, Lara M, Nguyen J, Kissel JT, Lossin C, Maselli RA. Defective fast inactivation recovery of Nav 1.4 in congenital myasthenic syndrome. Ann Neurol. 2015 May;77(5):840-50.

Burbidge SA, Daleb TJ, Powell AJ, Whitaker WRJ, Xie XM, Romanos MA, Clare JJ. Molecular cloning, distribution and functional analysis of the Nav 1.6 voltage-gated sodium channel from human brain. Molecular Brain Research 103 (2002) 80–90

Chatelier A, Dahllund L, Eriksson A, Krupp J, Chahine M. Biophysical properties of human Nav1.7 splice variante and their regulation by protein kinase A. J Neurophysiol 2008, 99: 2241-2250.

Cusdin FS, Nietlispach D, Maman J, Dale TJ, Powell AJ, Clare JJ, Jackson AP. The Sodium Channel β3-Subunit Induces Multiphasic Gating in NaV1.3 and Affects Fast Inactivation via Distinct Intracellular Regions. J Biol Chem 2010, 285 (43): 33404-33412.

Huang J, Yang Y, Zhao P, Gerrits MM, Hoeijmakers JG, Bekelaar K, Merkies IS, Faber CG, Dib-Hajj SD, Waxman SG. Small-fiber neuropathy Nav1.8 mutation shifts activation to hyperpolarized potentials and increases excitability of dorsal root ganglion neurons. J Neurosci. 2013 Aug 28; 33(35): 14087-97.

Misra SN, Kahlig KM, George AL Jr. Impaired NaV1.2 function and reduced cell surface expression in benign familial neonatal-infantile seizures. Epilepsia. 2008 Sep; 49(9): 1535-45.

Rhodes TH, Lossin C, Vanoye CG, Wang DW, George AL. Noninactivating voltage-gated sodium channels in severe myoclonic epilepsy of infancy. Proc Natl Acad Sci U S A. 2004 Jul 27; 101(30): 11147-52.

Vanoye CG, Kunic JD, Ehring GR, George AL Jr. Mechanism of sodium channel NaV1.9 potentiation by G-protein signaling. J Gen Physiol. 2013 Feb; 141(2): 193-202.

Zhang Z, Zhao Z, Liu Y, Wang W, Wu Y, Ding J. Kinetic model of Nav1.5 channel provides a subtle insight into slow inactivation associated excitability in cardiac cells. PLoS One. 2013 May 16; 8(5): e64286.
