# Supplementary material for: A single Markov-type kinetic model accounting for the macroscopic currents of all human voltage-gated sodium channel isoforms
Source: PLoS Comput Biol. 2017 Sep 1;13(9):e1005737. doi: 10.1371/journal.pcbi.1005737 (PMC5599066; doi:10.1371/journal.pcbi.1005737)
Supplement: S2 Appendix — (DOCX) [file pcbi.1005737.s002.docx]

**S2 Appendix. Examples of model channel implementation**

**Implementation in a morphologically reduced neuron model and comparison with s 14-state sodium Markov-type model.** The reduced neuron model by Maurice et al [1] reproduces the electrophysiological behaviour of a striatal cholinergic interneuron. It has a cylindric soma and two equivalent dendrities connected at the opposite sides of the soma, and it is equipped with 13 ionic channel mechanisms, built according to either the HH or Markov-type kinetic formalism. The sodium conductances are simulated, in particular, by means of two Markov-type kinetic models, to replicate respectively the fast and slow kinetics of the cellular sodium channels. The fast model of sodium channel is a Markov-type kinetic model supplied with 14 states (5 closed, one open, and 8 inactivated) and 36 transitions. More details can be found in the original paper [1].

S1 Fig reports the electrophysiological behaviour of this sodium channel model when examined by our virtual experimental protocols (see Fig 1 of the main text).

**S1 Fig. Electrophysiological features of a 14-state kinetic model of a fast sodium channel.** A: Voltage-clamp curves from -80 mV to 60 mV in step of 10 mV. B: Voltage dependence of the normalized conductance. C: Voltage dependence of normalized current during fast inctivation. D: Recovery from fast inactivation.

We replaced in the neuron model by Maurice et al [1] the fast sodium channel with our Na_V_1.2 channel, which was chosen because of its tissue distribution in the central nervous system [2], and of the overall similarity of its electrophysiological features with the original channel (S1 Fig).

The original neuron model exhibits autonomous spiking, even in the absence of synaptic inputs or external stimuli (S2 Fig A), reproducing the *in vivo* pacemaker discharge of its real counterpart. The fast sodium conductance is critical for such an electrophysiological behaviour, as can be observed when its conductance is set to zero (S2 Fig C). However, the insertion in the neuron model of our Na_V_1.2 VGSC is able to rescue the repetitive electrophysiological behaviour of the neuronal cell (S2 Fig B). After the insertion of our channel model, we only had to change the conductance densities at the soma (from 1 S/cm^2^ to 0.5 S/cm^2^) and dendrites (from 0.25 S/cm^2^ to 0.125 S/cm^2^), with all other parameters being left identical.

**S2 Fig. Comparison with a 14-state kinetic model of sodium channel.** A: Baseline autonomous spiking in a reduced neuron model of cholinergic striatal interneuron. B: After substitution of the original kinetic fast VGSC with the Na_V_1.2 model proposed, the model is able to reproduce the pacemaker discharge as well. C: Baseline activity in neuron model deprived by fast VGSC, all other parameters unchanged. D: Single action potential from the spiking train of the original model [1]. E: Single action potential after substituting the original fast sodium kinetic channel with the Na_V_1.2 VGSC of our model.

Moreover, according to our claim, the model bringing the Na_V_1.2 model, equipped with a lower number of states and transitions compared to the original one, completed the 5000 ms long run (S2 Fig 4A and C) in 2.87 s, against a 3.22 s duration of the simulation with the original model (integration time-step equal to 0.05 ms).

S2 Fig D and E show a zoomed action potential from the spontaneously occurring spike train in the original model and in that carrying our Na_V_1.2 model, respectively.

**Implementation in a morphologically detailed neuron model.** In previous work [3] we developed 14 morphologically detailed multi-compartmental conductance-based models of spinal alpha motoneuron, by taking advantage of an open-access web-based database of neuronal 3D morphologies, NeuroMorpho.org [4].

The digitized morphology of one of such neurocomputational model is shown in S3 Fig A. It comprises more than 3000 compartments and is equipped with six different ionic conductances, which vary in type and densities in the different sections (soma, dendrites, initial segment, axon) of the neuron model, as described in detail by the original paper [3].

**S3 Fig. Implementation in a morphologically detailed neuron model.** A: Digitized detailed 3D somato-dendritic morphology of a spinal motoneuron imported from NeuroMorpho.org [4] and implemented in a computational model [3]. B: Action potential evoked in the original model by an electrical impulse delivered at the soma. C: A similar spike obtained after substituting the original HH sodium channels with the Na_V_1.2 and Na_V_1.6 kinetic models.

The sodium conductances are provided by means of two mechanisms described in terms of the HH formalism for, respectively, a fast and a persistent sodium current. S3 Fig B shows the spike evoked at the initial segment and at the soma by an electrical stimulus 1 ms long with an intensity of 70 nA delivered to the soma.

S3 Fig C shows an action potential evoked by the same stimulus and recorded from the same locations, after substituting the original sodium channel with the kinetic channels presented in this study. We replaced the original fast sodium channel with the Na_V_1.2 model and the persistent one with the persistent version of the Na_V_1.6 model (see Fig 6C in the main text). We only needed to adjust for the maximal conductances of the substituted channels in different compartments, without changing the other parameters. In particular, no modification were made to the kinetics of the channels, nor to the active and passive properties, nor to the structural parameters of the neuron model.

Although a different distribution of real Na_V_1.2 and Na_V_1.6 channels in specialized subcellular structures has been discovered in experimental studies [5], we did not pass this knowledge in the current proof-of-concept implementation. However, the availability of a complete set of the different VGSC models, as those here provided, can allow to reproduce in detail and in a biologically inspired fashion also a segregation of different VGSCs in subcellular compartments, useful for experimentally testable simulations.

As expected, the time to complete the simulation with the HH formalism (S3 Fig B) was 10.28 s long, which increased to 16.47 s with the Markov-type channels (S3 Fig C) (integration time-step: 0.025 ms).

**References**

1. Maurice N, Mercer J, Chan CS, Hernandez-Lopez S, Held J, Tkatch T, et al. D2 dopamine receptor-mediated modulation of voltage-dependent Na^+^ channels reduces autonomous activity in striatal cholinergic interneurons. J Neurosci. 2004 Nov 17; 24(46): 10289-301.

2. Southan C, Sharman JL, Benson HE, Faccenda E, Pawson AJ, Alexander SP, et al. The IUPHAR/BPS Guide to PHARMACOLOGY in 2016: towards curated quantitative interactions between 1300 protein targets and 6000 ligands. Nucleic Acids Res. 2016 Jan 4; 44(D1): D1054-68.

3. Balbi P, Martinoia S, Massobrio P. Axon-somatic back-propagation in detailed models of spinal alpha motoneurons. Front Comput Neurosci. 2015 Feb 12; 9:15. doi: 10.3389/fncom.2015.00015.

4. Ascoli GA, Donohue DE, and Halavi M. NeuroMorpho.org: a central resource for neuronal morphologies. J. Neurosci. 2007; 27, 9247–9251. doi: 10.1523/JNEUROSCI.2055-07.2007.

5. Hu W, Tian C, Li T, Yang M, Hou H, Shu Y. Distinct contributions of Na(v)1.6 and Na(v)1.2 in action potential initiation and backpropagation. Nat Neurosci. 2009 Aug; 12(8): 996-1002. doi: 10.1038/nn.2359.
