# Supplementary figures and images for: A single Markov-type kinetic model accounting for the macroscopic currents of all human voltage-gated sodium channel isoforms
Source: PLoS Comput Biol. 2017 Sep 1;13(9):e1005737. doi: 10.1371/journal.pcbi.1005737 (PMC5599066; doi:10.1371/journal.pcbi.1005737)

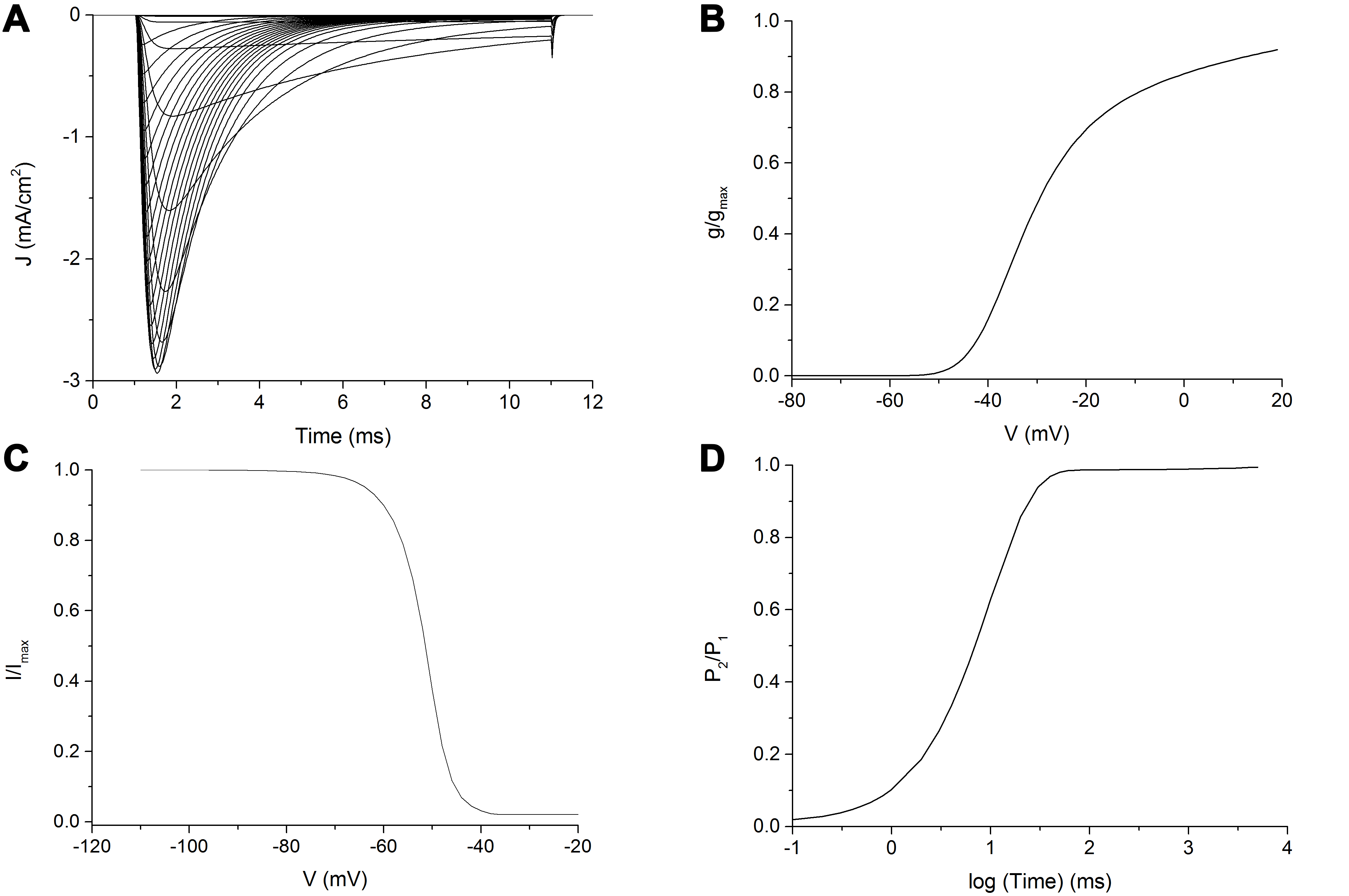

Supplement: S1 Fig — A: Voltage-clamp curves from -80 mV to 60 mV in step of 10 mV. B: Voltage dependence of the normalized conductance. C: Voltage dependence of normalized current during fast inctivation. D: Recovery from fast inactivation. (TIF) [file pcbi.1005737.s003.tif]

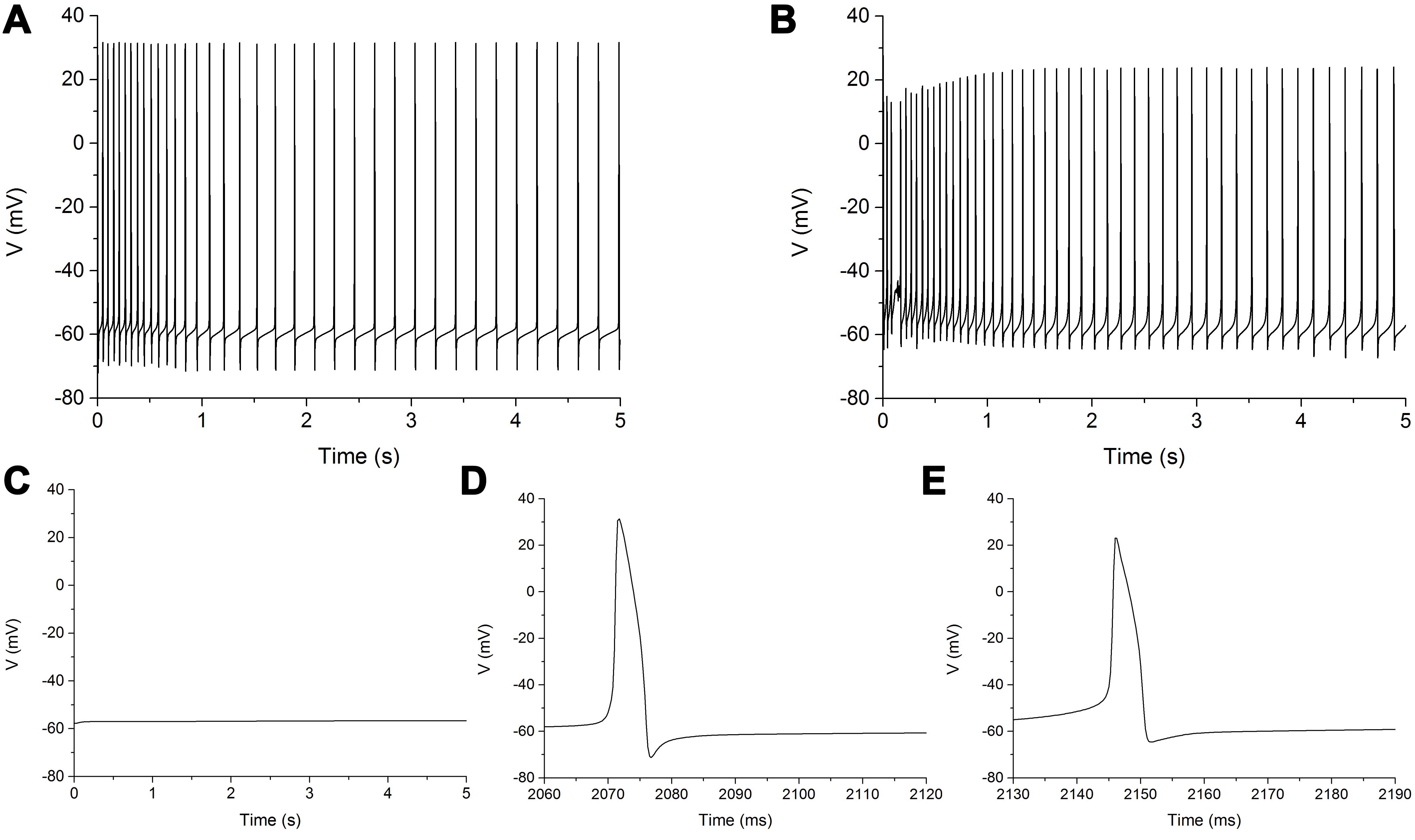

Supplement: S2 Fig — A: Baseline autonomous spiking in a reduced neuron model of cholinergic striatal interneuron. B: After substitution of the original kinetic fast VGSC with the NaV1.2 model proposed, the model is able to reproduce the pacemaker discharge as well. C: Baseline activity in neuron model deprived by fast VGSC, all other parameters unchanged. D: Single action potential from the spiking train of the original model [1]. E: Single action potential after substituting the original fast sodium kinetic channel with the NaV1.2 VGSC of our model. (TIF) [file pcbi.1005737.s004.tif]

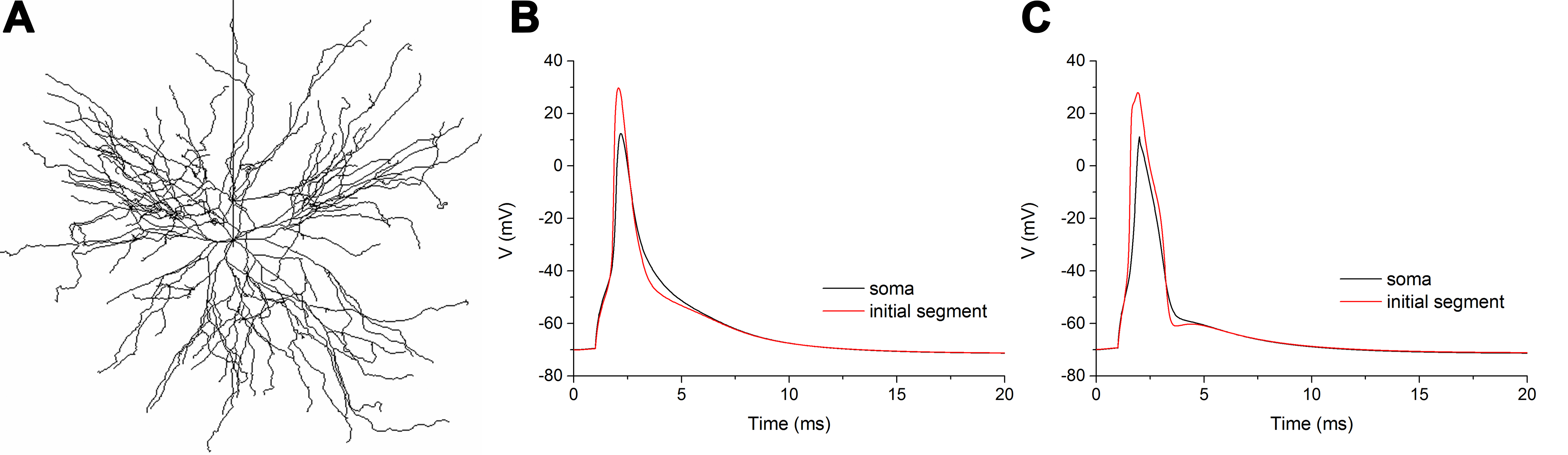

Supplement: S3 Fig — A: Digitized detailed 3D somato-dendritic morphology of a spinal motoneuron imported from NeuroMorpho.org [4] and implemented in a computational model [3]. B: Action potential evoked in the original model by an electrical impulse delivered at the soma. C: A similar spike obtained after substituting the original HH sodium channels with the NaV1.2 and NaV1.6 kinetic models. (TIF) [file pcbi.1005737.s005.tif]
